# Unveiling Emerging Opportunistic Fish Pathogens in Aquaculture: A Comprehensive Seasonal Study of Microbial Composition in Mediterranean Fish Hatcheries

**DOI:** 10.3390/microorganisms12112281

**Published:** 2024-11-10

**Authors:** Dimitrios Skliros, Maria Kostakou, Constantina Kokkari, Maria Ioanna Tsertou, Christina Pavloudi, Haris Zafeiropoulos, Pantelis Katharios, Emmanouil Flemetakis

**Affiliations:** 1 Laboratory of Molecular Biology, Department of Biotechnology, School of Applied Biology and Biotechnology, Agricultural University of Athens, 11855 Athens, Greece; dsklhros@gmail.com (D.S.);; 2 Department of Applied Microbial Ecology, Helmholtz Centre for Environmental Research—UFZ, 04318 Leipzig, Germany; 3 Institute of Marine Biology, Biotechnology and Aquaculture, Hellenic Centre for Marine Research, 71500 Heraklion, Greece; dkok@hcmr.gr (C.K.); tsertou@hcmr.gr (M.I.T.); katharios@hcmr.gr (P.K.); 4 European Marine Biological Resource Centre—European Research Infrastructure Consortium (EMBRC-ERIC), 75252 Paris, France; christina.pavloudi@embrc.eu; 5 Laboratory of Molecular Bacteriology, Department of Microbiology, Immunology and Transplantation, Rega Institute for Medical Research, KU Leuven, 3000 Leuven, Belgium; haris.zafeiropoulos@kuleuven.be

**Keywords:** microbial community, metagenomics, microbial ecology, bacteriome, environmental microbiology

## Abstract

The importance of microbial communities in fish hatcheries for fish health and welfare has been recognized, with several studies mapping these communities during healthy rearing conditions and disease outbreaks. In this study, we analyzed the bacteriome of the live feeds, such as microalgae, rotifers, and *Artemia*, used in fish hatcheries that produce Mediterranean species. Our goal was to provide baseline information about their structure, emphasizing in environmental putative fish pathogenic bacteria. We conducted 16S rRNA amplicon Novaseq sequencing for our analysis, and we inferred 46,745 taxonomically annotated ASVs. Results showed that incoming environmental water plays a significant role in the presence of important taxa that constitute presumptive pathogens. Bio-statistical analyses revealed a relatively stable bacteriome among seasonal samplings for every hatchery but a diverse bacteriome between sampling stations and a distinct core bacteriome for each hatchery. Analysis of putative opportunistic fish pathogenic genera revealed some co-occurrence correlation events and a high average relative abundance of *Vibrio*, *Tenacibaculum*, and *Photobacterium* genera in live feeds, reaching a grand mean average of up to 7.3% for the hatchery of the Hellenic Center of Marine Research (HCMR), 12% for Hatchery A, and 11.5% for Hatchery B. Mapping the bacteriome in live feeds is pivotal for understanding the marine environment and distinct aquaculture practices and can guide improvements in hatchery management, enhancing fish health and sustainability in the Mediterranean region.

## 1. Introduction

The significance of the bacteriome in fish hatcheries for maintaining fish health and welfare has been acknowledged, with numerous studies mapping these communities under both healthy rearing conditions and during disease outbreaks [1,2]. Modern fish hatcheries are areas of high biosecurity, and strict protocols are applied to prevent the entry of microbial pathogens [3,4,5]. Fish in their early stages of development are particularly susceptible to infectious diseases because they do not have a fully developed immune system [6]. Additionally, the existing rearing methodologies rely on live feeds, such as microalgae, rotifers, and *Artemia*, for a significant period, typically starting at 4 days post-hatching (dph) and ending at 40 dph, depending on the species reared [7,8]. Despite the strict biosecurity protocols, the entry of pathogens into the rearing tanks through a live feed is inevitable. Thus, these first days of rearing constitute a critical time window, during which significant mortality due to bacterial infections can occur [9].

In addition to the risk of disease, this time window is crucial for the development of a healthy bacteriome in the intestines of fish larvae, which is an invaluable asset for their subsequent transition to the on-growing stage in the open sea, as the intestinal bacteriome is linked to growth and health performance [10]. Therefore, knowledge and regulation of the microbial communities of live feeds should not only be seen as a means for preventing the occurrence of diseases but also as an opportunity to redefine the quality standards of the juveniles produced [11].

Microbial relationships and interdependencies in the hatchery environment are complex and poorly understood despite their critical importance in fish health and welfare. Several scientific articles have highlighted this importance in the past [12,13]. This complexity is particularly evident in marine fish hatcheries, where live feeds are produced in separate infrastructures, which, in some cases, are able to interact before being administered to fish [14]. For example, microalgae can be used directly in fish larvae tanks (using the “green water” technique) and can also be used as a feed for rotifers [15]. Additionally, each commercial hatchery may use different strains of live feeds, particularly microalgae, as well as different culture techniques that directly affect the composition and diversity of host-associated microbial communities.

Host-associated microbial communities exhibit extensive plasticity, determined by both the host and the environment, and can reflect previous processes. More specifically, the rearing factors’ procedures and sequence can have a detrimental role on which and when specific bacterial taxa in a community will affect its structure through colonization/niche occupation and competition with other microbes [16]. It is well established that only a small fraction of environmental bacteria can be cultured in vitro, making a comprehensive analysis difficult. In recent years, this obstacle has been largely overcome with the advent of new technologies, such as high-throughput sequencing. This approach enables the accurate profiling of microbial diversity in environmental samples down to the genus level without the need for isolating and culturing microorganisms [17].

Our study aimed to use a deep sequencing pipeline to provide crucial baseline information on the structure and diversity of microbial communities in marine hatcheries. This information is critical for optimizing the bacteriome modulation of fish larvae, ultimately leading to the production of high-quality juveniles. The primary objective of the study was to assess how differences in rearing environments and techniques, as well as in the water source, influence the microbial composition of the hatchery, with a particular focus on live feeds. An emphasis on emerging major environmental opportunistic fish pathogenic bacteria and seasonal correlation also took place.

## 2. Materials and Methods

### 2.1. Samples Overview and Hatcheries Involved in the Study

**Figure 1:**
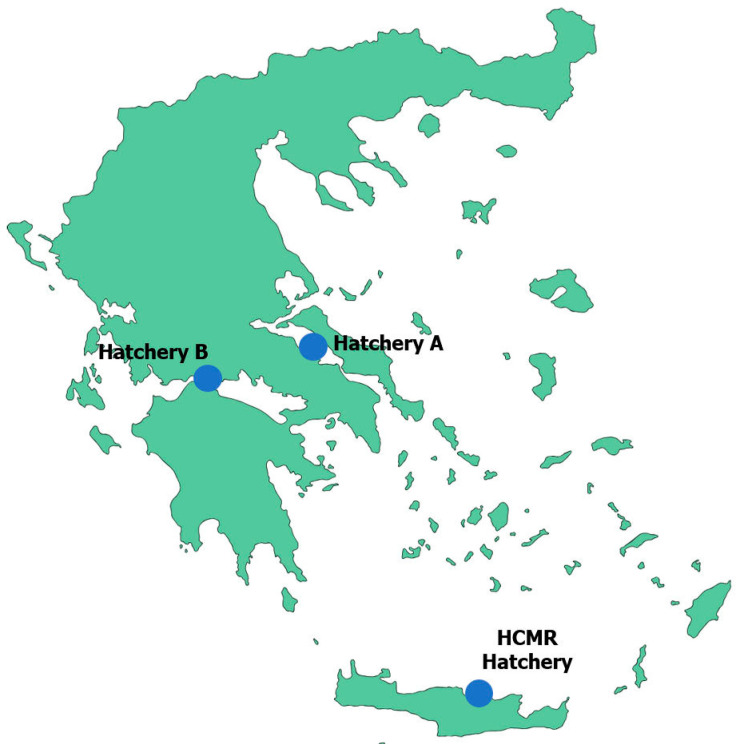
Geographic location of fish hatcheries. Brief map of Greece and geographical distribution of the fish hatcheries reported in the present study.

Three Mediterranean marine fish hatcheries located in different regions of Greece were selected for this study. They were chosen based on their unique water sources and treatments, as well as their varying levels of intensity in rearing practices (Hellenic Center of Marine Research (HCMR) hatchery, Hatchery A, Hatchery B; Figure 1; Table 1 and Table 2).

We utilized state-of-the-art culture-independent techniques to thoroughly analyze and track the bacterial communities of live-feed sections over the course of one year. By conducting a comparative bacteriome analysis, we were able to closely follow the dynamics of the bacterial communities present in the live feeds of the marine fish hatcheries, with a particular focus on putative pathogenic opportunistic bacteria, particularly those belonging to the *Vibrio* and *Tenacibaculum* genera [18], which are known to include significant disease agents for fish larvae. Sampling was carried out seasonally and in triplicates during Autumn, Winter, and Spring. The sampling process involved collecting water samples from five different areas within each hatchery. These areas included inlet water (24 samples), microalgae (27 samples), rotifers (27 samples), *Artemia* (24 samples), and outlet water (27 samples). *Artemia* samples from the HCMR hatchery were absent during Winter, as well as inlet water samples during Autumn from Hatchery B, meaning the total number of analyzed samples was 129. In total, 42 samples were analyzed for the HCMR hatchery, 45 samples for Hatchery A, and 42 samples for Hatchery B. The number of samples in each sampling point per hatchery is presented in detail in Figure S1. During our monitoring program, no fish mortality events due to bacterial pathogens were reported.

### 2.2. Sampling Method

Microbial populations were concentrated from water samples using an updated protocol of isolating environmental DNA [19,20]. Water samples were collected in triplicates into sterile plastic bottles and preserved at 4 °C for a short time. Sample water from the live feeds was filtered through a fine mesh to remove the planktonic organisms, followed by sorvall centrifugation at 500× *g* rpm for 2 min to remove any residues of the live feeds. For each sample, at least 2.5 L of water was collected. Following collection, samples were filtered through a 25 mm diameter, 0.2 μm pore size polycarbonate filter (Whatman plc, Maidstone, UK) using a vacuum filtration system. The filters were then transferred to cryovials and stored at −80 °C until assayed. All glassware and collection bottles were cleaned before use with a 2% bleach solution, followed by thorough washing with ethanol and sterile water.

### 2.3. DNA Extraction and 16S rRNA Sequencing

Total DNA was extracted using an optimized SDS-based extraction buffer and phenol/chloroform-based extraction method [21]. The extraction buffer contained 10 mM Tris (pH 7.2), 0.1 mM EDTA, 2% SDS, b-mercaptoethanol, and proteinase k (Thermo Fisher, Waltham, MA, USA). Filters were homogenized with liquid nitrogen before adding an extraction buffer. Samples were then incubated for 2.5 h at 56 °C. The liquid phase containing nucleic acids was separated with centrifugation at 11,000× *g* for 15 min at 4 °C. Total nucleic acids were purified twice by using an equal volume of phenol: chloroform: isoamyl alcohol (25:24:1) before precipitation from the aquatic phase with isopropanol overnight at −20 °C. Total nucleic acids were washed with absolute and 70% ethanol and resuspended in ddH_2_O. An RNAse (Qiagen, Hilden, Germany) step was followed at 37 °C for 1 h, and DNA was purified with a commercial DNA isolation kit (Macherey-Nagel, Düren, Germany) according to the manufacturer’s protocol. The yield was quantified using a Nanodrop spectrophotometer (Thermo Fisher, Waltham, MA, USA). DNA quality was estimated with 0.7% agarose gel.

A total of 50 ng of DNA was amplified following the 16S Metagenomic Sequencing Library Illumina protocol. In summary, in the first amplification step, primers were designed containing (1) a universal linker sequence, allowing amplicons for incorporation indexes and sequencing primers by a Nextera XT Index kit (Illumina, San Diego, CA, USA); (2) 16S rRNA gene universal primers, amplifying the 16s rRNA region V3–V4 [22]. In the second and last assay, amplification indexes were included. Amplicon libraries were quantified by fluorimetry using the Quant-iT™ PicoGreen™ dsDNA Assay Kit (Thermo Fisher, Waltham, MA, USA). Libraries were pooled prior to sequencing on the NovaSeq 6000 platform system (Illumina, San Diego, CA, USA) in 250 cycles of paired reads configuration. The size and quantity of the pool were assessed on the Bioanalyzer 2100 (Agilent, Santa Clara, CA, USA) and with the Library Quantification Kit for Illumina (Kapa Biosciences, Wilmington, MA, USA), respectively. The PhiX Control library (v3) (Illumina, San Diego, CA, USA) was combined with the amplicon library (expected at 20%). Sequencing data were available within approximately 56 h and with an approximate size of 6 gigabytes.

### 2.4. Bioinformatics and Bio-Statistics Analysis

Image analysis, base calling, and a data quality assessment were performed on the NovaSeq instrument (NovaSeq Control Software (NVCS version 1.7)). Raw sequences were imported into the QIIME2 platform [23]. Cutadapt version 3.4 plugin was used to filter specific V3–V4 16S rRNA region adapters. Reads were processed using the ‘denoise-paired’ command of the DADA2 plugin [24]. Low-quality reads were filtered by the function ‘filterAndTrim’ and were truncated where they started to lose quality (240 bp for R1, 200 bp for R2). Error models were generated using the ‘learnErrors’ function, and the DADA2 algorithm was applied using the ‘dada’ function. ASVs (‘Amplicon Sequence Variants’) generated by R1 and R2 reads were merged using the ‘mergepairs’ function. ASVs analysis is recommended as an optimal method with the highest comprehensiveness method for discriminating species, with 99% grouping sequence similarity [25]. Chimeric sequences were removed using the ‘removeChimeraDenovo’ function. Taxonomy of resulting ASVs was annotated using blastn v2.2.29+ [26] against a 16S rRNA-specific database from the NCBI (version August 2021). Assigned taxonomies with an identity percentage lower than 97% were reassigned using the NBAYES algorithm [27] against SILVA v.138. Data were normalized using rarefaction [28] in the phyloseq R package [29] to perform an alpha diversity analysis. Shannon, Simpson, and Richness indexes were calculated using the vegan R package (version 2.5-4) [30], and the Wilcox test was used to find significant differences in alpha diversity between the groups. Pearson’s correlation analysis of opportunistic fish pathogenic genera and ANOVA statistical significance analysis of the average relative abundance of bacterial genera (post hoc test LSD) took place with SPSS (version 26.0; IBM, Armonk, NY, USA). Visualization of ASVs and the average relative abundance of pathogenic opportunistic fish took place with the Sigma plot (version 14.0; Graffiti LLC, Irvine, CA, USA).

To estimate the potential sources of microbes in the outlet water, Sourcetracker (version 1.0.1) was used [31] on the averages of the replicate samples under different scenarios: (a) Sources: inlet water, microalgae, rotifers, *Artemia*, Sink: outlet water, (b) Sources: inlet water, microalgae, Sink: rotifer. These scenarios were run for the subset of the putative pathogen ASVs (species of the *Vibrio*, *Tenacibaculum*, *Alivibrio*, and *Photobacterium* genera). In addition, FlashWeave (version 0.19) [32] was used to infer associations between ASVs in Hatchery B during Winter. All further data analysis was carried out using the R statistical software version 4.2.0. Alpha diversity analysis (Chao1, Shannon, and Simpson), core microbiome analysis (detection threshold: 0.001 and prevalence threshold: 0.3), and visualization of prevalence distributions were performed using the microbiome R package (v1.19.0) [33]. Classification plots were done using a phyloseq package (v1.41.0) [29] and principal component analysis (PCA) and using the R stats package (v.3.6.2).

## 3. Results

### 3.1. Sequencing, Quality Filtering, and Samples Richness

A total of 129 samples were sequenced using paired-end sequencing, generating 77,054,222 reads with an average of 298,660 reads per sample, an average file size of 74.8 megabytes per sample, an average length of 250.7 base pairs, and an average quality score of 34.6. After quality filtering, 21,008,815 (~27%) high-quality sequences were retained, with an average of 162,859 sequences per library. The 46,745 annotated amplicon sequence variants (ASVs) represented an average of 362 ASVs per sample. Rarefaction analysis indicated that all samples reached a plateau phase at both genus and ASV levels, suggesting that additional sequencing would not largely increase the number of annotated taxa or ASV (Figure S2). In terms of taxonomy, we identified 51 phyla, 322 families, and 801 genera across all hatcheries. Unmapped and unknown reads accounted for 17.5 ± 1.6% (mean ± SE) at the family level and 2.7 ± 0.77% (mean ± SE) at the genus level of the total reads. The richness of samples differed significantly among the three hatcheries across all seasons. Chao1 richness analysis revealed that the HCMR hatchery had an average value of 30.05 (SE ± 4.01) at the genus level and 32.52 (SE ± 3.23) at the ASVs level. Hatchery A had an average value of 31.34 (SE ± 3.06) at the genus level and 29.49 (SE ± 2.73) at the ASVs level. Finally, Hatchery B had the highest average value, with 43.46 (SE ± 3.71) at the genus level and 44.24 (SE ± 3.98) at the ASVs level, indicating a greater richness of bacterial taxa compared to the other hatcheries. In general, no important human pathogenic bacterial genera were detected in our analysis, with the exception of the *Pseudomonas* genus.

### 3.2. Diversity, Prevalence, and Core Bacteriome of the Live-Feed Cultures Bacteriome

**Figure 2:**
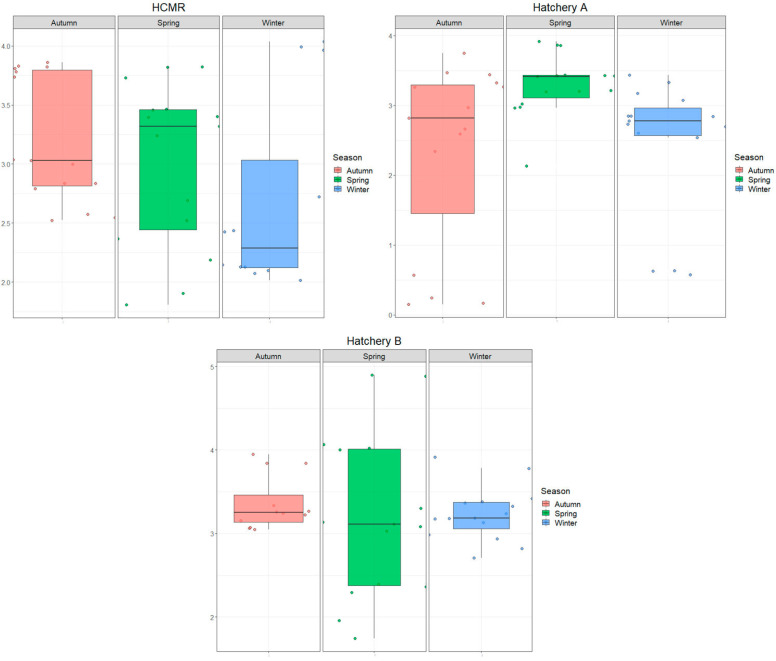
Seasonal variation of bacterial genera. Shannon index of the three hatcheries (HCMR hatchery, Hatchery A and Hatchery B). Microbiomes’ diversity has been grouped according to seasonal effect.

**Figure 3:**
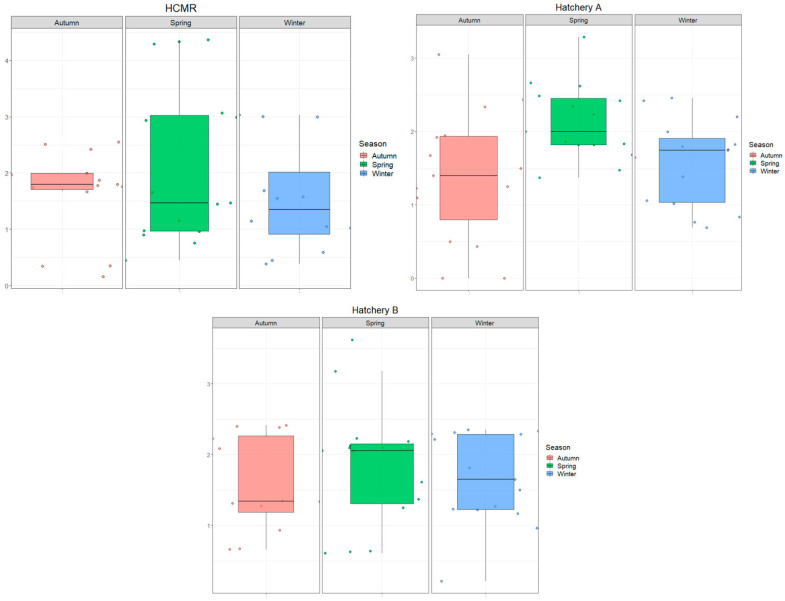
Seasonal variation of bacterial ASVs. Shannon index of the three hatcheries (HCMR hatchery, Hatchery A and Hatchery B). Microbiomes’ diversity has been grouped according to seasonal effect.

Principal component analysis (PCA) was conducted separately for each hatchery, using the most abundant and common genera, and low deviations were observed among triplicate samples. In HCMR, 9 genera were common across all three live-feed sections during all sampling seasons, 13 genera in Hatchery A, and 18 in Hatchery B. PCA was used to monitor the beta-diversity among the samples of each hatchery and identify the principal genera that contribute to the diversity of the bacteriome (Figures S3 and S4). By inserting the variables independently and grouping them according to live-feed type and season, it was observed that triplicates had low variability and were grouped separately depending on the live-feed sampling site (rotifers, *Artemia*, microalgae). However, the seasonal effect increased the deviation of the bacteriome variability in all hatcheries. PC1 and PC2 were sufficient in distinguishing the bacteriomes of the live feeds for each season, with an aggregated contribution of 54.97% for HCMR (Figure S3A), 45.2% for Hatchery A (Figure S3B), and 44.37% for Hatchery B (Figure S3C). This suggests that seasonality could separate the live feed bacteriomes, mostly in HCMR and Hatchery B, although further exploration is needed to determine the significance of this bacteriome shift. The PCA loadings identified the primary genera contributing to the variation in live feeds across all seasons for all hatcheries (Figure S4). More specifically, for HCMR hatchery, genera such as *Vibrio*, *Tenacibaculum*, *Alteromonas*, *Phaeobacter*, and *Pseudomoalteromonas* contributed the most to the variation among live feeds and seasons. In Hatchery A, the top contributing genera for the variation of the samples were *Vibrio*, *Tenacibaculum*, *Alteromonas*, *Pseudomoalteromonas*, *Marinobacter*, *Pseudomonas*, *Clacieola*, and *Polaribacter*, while in Hatchery B, the genera were Vibrio, *Tenacibaculum*, *Alteromonas*, *Pseudomoalteromonas*, *Maribacter*, *Tritonibacter*, *Rosevarius*, *Brumimicobrium*, and *Muricauda*. To determine the significance of seasonal bacteriome shifts in terms of bacterial richness, we used alpha diversity metrics (Figure 2), which pinpoint the richness of different genera and species in the various hatcheries, including the bacteriomes of the inlet and outlet water. The Shannon index revealed that the sampling season had a statistically significant effect on rare occasions, with higher values observed at HCMR, indicating higher bacteriome diversification compared to the other hatcheries (Figure 2). Moreover, the Shannon index showed that the season had little effect on the genera variability of the HCMR hatchery, with values ranging between 0.5 and 3.00 and *p*-values of 0.183, 0.777, and 0.263 when comparing Winter with Spring, Winter with Autumn, and Spring with Autumn, respectively. At Hatchery A (Figure 2), Shannon index values ranged between 0.4 and 4.3, indicating a similar genus diversification. However, values differed among seasons, with a statistically significant increase observed during the Spring season (*p* = 0.005 compared to Winter and *p* = 0.032 compared to Autumn), indicating a significant increase in bacteriome diversity during this time. We did not observe a statistically significant difference between Winter and Autumn (*p* = 0.457). Finally, Hatchery B (Figure 2) had Shannon index values ranging from 0.2 to 3.6, with no statistically significant difference among seasons (*p* = 0.477, 0.815, and 0.367 when comparing Winter with Spring, Winter with Autumn, and Spring with Autumn, respectively), aligning with the low genera fluctuation observed at the HCMR hatchery, despite the higher richness values. The Shannon index indicated that alpha diversity at the species level was relatively consistent among the three hatcheries studied, with values ranging from 1.8 to 4.0 (Figure 3). The impact of season on ASVs diversity within each hatchery was found to be minimal according to statistical analysis. Specifically, there were no significant differences in bacteriome diversity between seasons in the HCMR hatchery (Figure 3), with *p*-values of 0.222, 0.056, and 0.445 when comparing Winter with Spring, Winter with Autumn, and Spring with Autumn, respectively. Hatchery A (Figure 3) showed similar values to the genus level, ranging from 0.5 to 3.9. A statistically significant increase was observed from Spring to Winter (*p* = 0.024) and from Spring to Autumn (*p* = 0.011), similar to what was observed at the genus level. However, the comparison between Winter and Autumn remained unaffected (*p* = 0.756). Finally, Hatchery B (Figure 3) had values between 1.7 and 4, which were consistent with the genus-level results, and there was no statistically significant difference in diversity among seasons (*p* = 0.942, 0.627, and 0.579 when comparing Winter with Spring, Winter with Autumn, and Spring with Autumn, respectively), following a similar pattern to the genus-level diversity. The bacteriome composition of all three hatcheries was dominated by Proteobacteria and Bacteroidetes, as shown in Figure S5. Additionally, Actinobacteria and Firmicutes were also present, albeit in smaller proportions. Interestingly, the prevalence of Firmicutes and Actinobacteria varied among the hatcheries, with the HCMR hatchery having a higher prevalence of Actinobacteria than Firmicutes, while Hatchery A and B showed an opposite trend. The core bacteriome of the hatcheries, which is presented in Table 3, was found to be diverse. Hatchery A and B had 12 and 13 genera, respectively, that were present across all sampling stations and seasons. The HCMR hatchery had nine genera that were part of its core bacteriome. Many of these core microbiotas belonged to typical Mediterranean bacteria genera, such as *Alteromonas*, *Pseudoalteromonas*, and *Vibrio*. Hatchery B, which uses natural seawater, had additional genera in its core bacteriome, such as *Marinomonas*, *Catenococcus*, and *Maribacter*. Although opportunistic fish pathogenic bacteria genera, such as Vibrios, were present in all three core bacteriomes, the *Tenacibaculum* genus was only found in the core bacteriome of Hatchery B.

### 3.3. Relative Abundance of the Predominant Marine Bacteria in the Live-Feed Cultures

**Figure 4:**
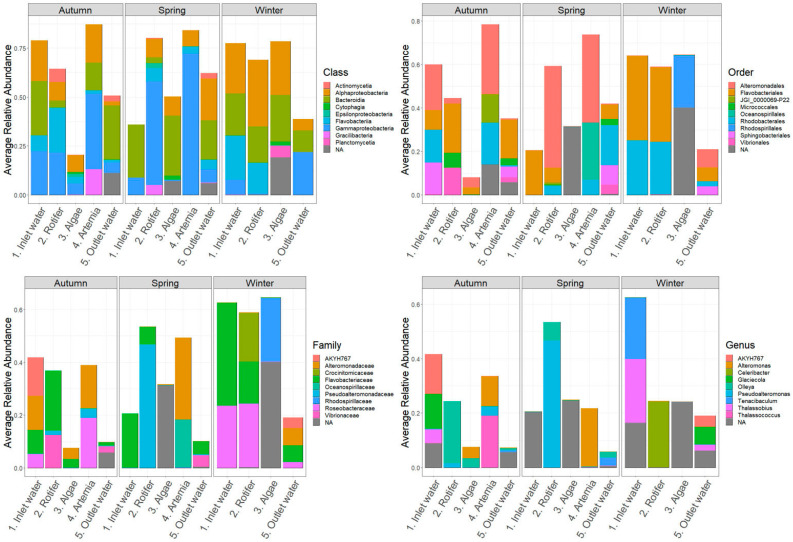
Ten most abundant Classes, Orders, Families and Genera of the HCMR hatchery. Average relative abundances of the 10 most abundant Classes, Orders, Families and Genera of the HCMR hatchery per season and per sampling point.

Figure 4 presents the average relative abundances of bacterial classes in HCMR hatchery across different sampling stations and seasons. The dominant classes were Gammaproteobacteria (10–65%), Alphaproteobacteria (10–25%), Flavobacteria (5–15%), and Actinomycitia (2–15%). Gammaproteobacteria were the most abundant across all samples, while Alphaproteobacteria were primarily observed in *Artemia* and outlet water samples. Flavobacteria were most abundant in microalgae live-feed samples during the Spring season. At the order level, Alteromonadales, Flavobacteriales, Vibrionales, and Rhodobacteriales were consistently present in all samples throughout the three seasons. Family-level abundance analysis revealed higher diversification, represented mainly by *Vibrionaceae*, *Flavobacteriaceae*, and *Alteromonadaceae* families only present in microalgae samples during Autumn, inlet water during Winter and Autumn, and microalgae samples during Winter, respectively.

**Figure 5:**
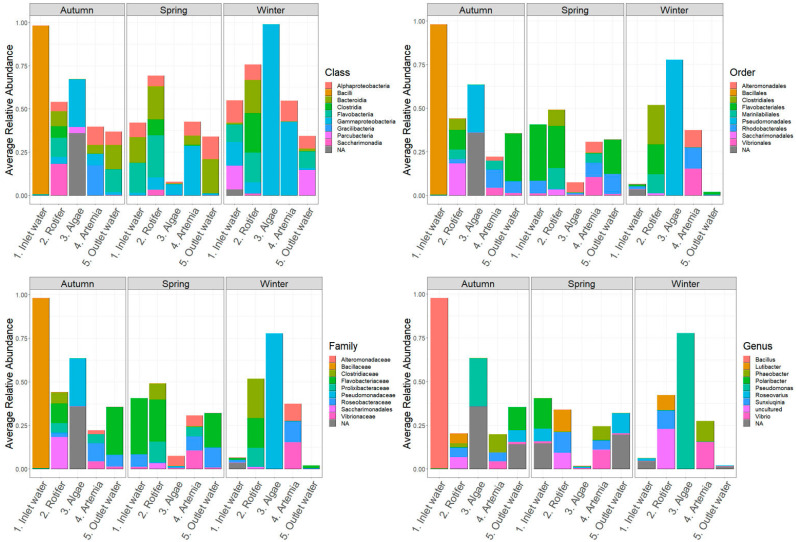
Ten most abundant Classes, Orders, Families and Genera of Hatchery A. Average relative abundances of the 10 most abundant Classes, Orders, Families and Genera of Hatchery A per season and per sampling point.

In Hatchery A, Gammaproteobacteria (5–95%), Alphaproteobacteria (5–25%), and Bacteriodia were the main classes observed across all samples and seasons (Figure 5), which is different from the HCMR hatchery. Vibrionales, Rhodobacteriales, and Flavobacteriales were the dominant orders. The *Pseudomonadaceae* family appeared with significant abundance in microalgae samples during Autumn and Spring. *Vibrio* and *Roseovarius* genera are prevalent across all samples and seasons. However, during Winter and Autumn, the *Pseudomonas* genus dominated the microalgae live-feed samples. Two distinct ASVs were grouped with annotation analysis, showing that *Pseudomonas* ASV77, with an average relative abundance of 21.1%, and *Pseudomonas* ASV14, with an average relative abundance of ~77.5%, were the two suspected dominant species during Winter. During Autumn, annotation of the two dominant ASVs identified *Pseudomonas* ASV89, with an average relative abundance of 27.3%, and *Pseudomonas* ASV320, with an average relative abundance of 5%. Similar to Autumn on inlet water, *Alteromonas* genera also showed a significant relative abundance in Hatchery A. Finally, ASV4, which was annotated within *Bacillus* sp., had a significant presence in the bacteriome of the live feed, such as a *Bacillus* sp. (ASV analysis annotated it as *Bacillus mobilis*) in inlet water during the Autumn season, which constituted an average relative abundance of more than ~97% of the total bacteriome of the sample, indicating a possible contamination event.

**Figure 6:**
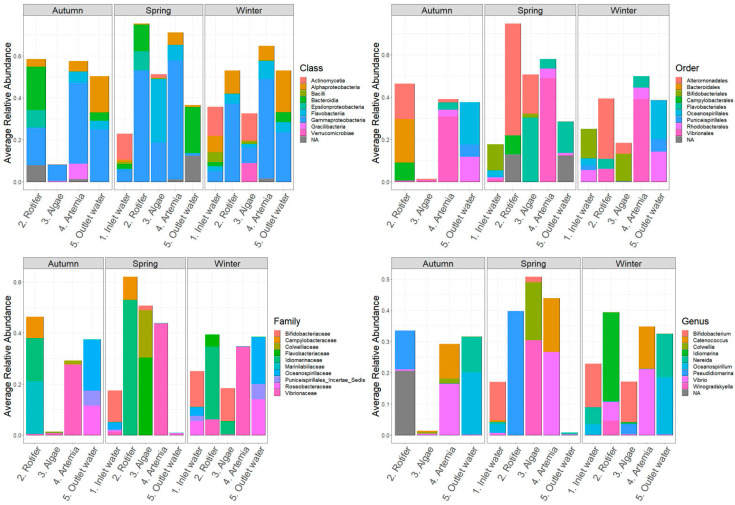
Ten most abundant Classes, Orders, Families and Genera of Hatchery B. Average relative abundances of the 10 most abundant Classes, Orders, Families and Genera of Hatchery B per season and per sampling point.

In Hatchery B, the microbial composition of live feeds showed Gammaproteobacteria (5–55%) and Flavobacteria (2–30%) as the main classes (Figure 6). Among thedepicted orders, Alteromonadales (10–50%), especially in rotifer samples, and Vibrionales, which reached up to 55% in *Artemia* samples, were the most abundant. At the family level, *Vibrionaceae* and *Alteromonadaceae* showed a similar pattern of abundance, while *Roseobacteriaceae*, *Oceanospiriliceae*, and *Monodopsidaceae* together reached up to 50% in outlet water during Autumn and Winter. The most prevalent genera were *Vibrio*, *Alteromonas*, and *Pelagimonas*.

### 3.4. Correlation of Putative Opportunistic Fish Pathogenic Bacteria with Sampling Points and Hatcheries

**Figure 7:**
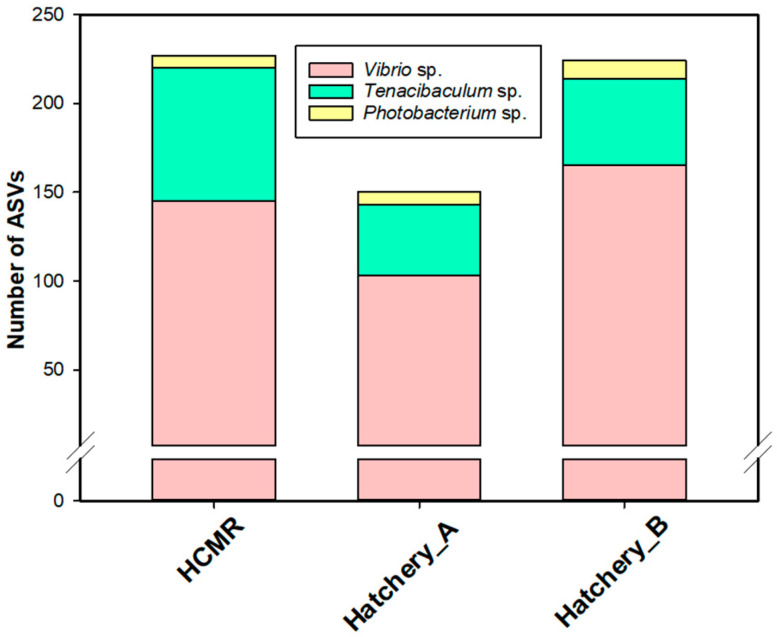
ASVs of opportunistic fish pathogenic genera. Number of ASVs representing selected opportunistic fish pathogenic genera across all three studied hatcheries.

**Figure 8:**
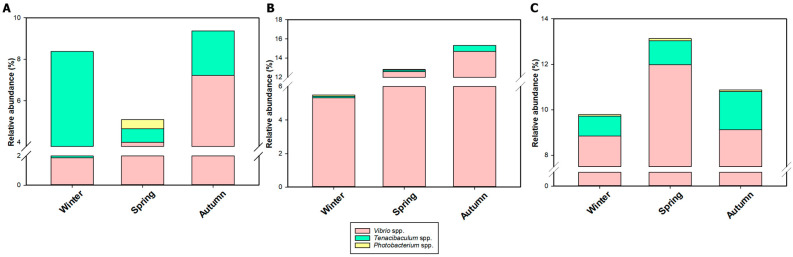
Most abundant opportunistic fish pathogenic genera. Average relative abundance of opportunistic fish pathogenic genera across the three seasons and all three studied hatcheries ((**A**) for HCMR hatchery, (**B**) for Hatchery A and (**C**) for Hatchery B).

An analysis was performed to specifically focus on the presence of putative fish pathogens, which was narrowed to species belonging to *Vibrio*, *Tenacibaculum*, and *Photobacterium* genera. In the HCMR hatchery, we identified 145, 75, and 7 ASVs associated with the genera *Vibrio*, *Tenacibaculum*, and *Photobacterium*, respectively. Furthermore, Hatchery A exhibited 103, 40, and 7 ASVs for the genera *Vibrio*, *Tenacibaculum*, and *Photobacterium*, respectively, while Hatchery B showed 165, 49, and 10 ASVs for the same genera (Figure 7). Additionally, as a result of the analysis, we present the grand mean of the average relative abundance of each opportunistic fish pathogenic genera separately for all seasons (Figure 8; Supplementary Data) as well the first annotation result of ASVs that correspond to presumably opportunistic fish pathogenic taxa (Tables S1–S3). At the HCMR hatchery, the average relative abundance of bacteria belonging to these genera was 7.75%, with *Vibrio* spp. representing 4.7%, *Tenacibaculum* spp. representing 2.6%, and *Photobacterium* spp. representing 0.45%. In Hatchery A, the average relative abundance of putative fish pathogens was 12.12%, with *Vibrio* spp. representing 11.7%, *Tenacibaculum* spp. representing 0.3%, and *Photobacterium* spp. representing 0.12%. In Hatchery B, the average relative abundance of putative fish pathogens was 11.7%, with *Vibrio* spp. representing 10.2%, *Tenacibaculum* spp. representing 1.3%, and *Photobacterium* spp. representing 0.2%. In the case of sampling points (Supplementary Data) and the HCMR hatchery, opportunistic pathogenic bacterial genera were primarily present in the hatchery inlet water (~9.5%) and less in outlet water (~7.9%). In the case of live feeds, *Vibrio* spp. ASVs were detected at a significant percentage in all live-feed samples (3.5–7.2%), contrary to inlet and outlet water, whereas *Tenacibaculum* spp. was more abundant. *Photobacterium* spp. were solely reported in inlet water (~0.7%).

**Figure 9:**
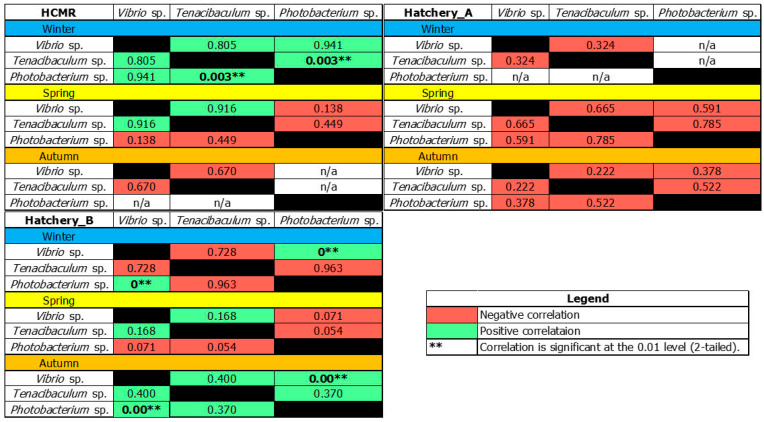
Spearman’s correlation analysis of opportunistic fish pathogenic genera. Co-occurrence Spearman’s correlation analysis of most abundant opportunistic fish pathogenic genera with their respected *p* values. n/a for not available.

In Hatchery A, the relative abundance of the *Tenacibaculum* genus was significantly lower across all sampling points compared to the HCMR hatchery. *Vibrio* genus was reported in all sampling points, with the highest being in rotifer samples, reaching 36%. Inlet and outlet water had an 11 and 3% average relative abundance, respectively. *Tenacibaculum* genus was reported in *Artemia* (~1%) and in outlet water (~0.5%). *Photobacterium* genus was detected similarly in rotifer and microalgae samples (~0.1%). Finally, regarding Hatchery B, Vibrios appeared abundant across all sampling points, with the highest abundance being 31% in *Artemia* samples. *Tenacibaculum* spp. was present in both outlet and inlet water, with an average relative abundance of ~2%, while it was also detected in *Artemia* samples (~1.2%). Finally, the *Photobacterium* genus was detected only in Artemia samples (~0.3%). These results prompt us to perform a Spearman’s correlation analysis between the abundance of the putative pathogenic genera and sampling seasons for each Hatchery separately. This way, we can identify the possible co-occurrence of the abundance of presumably fish pathogenic genera in a specific period (Figure 9). In the case of the HCMR hatchery, a positive correlation among all studied genera was reported during the Winter season, with a statistically significant positive correlation of *Tenacibaculum* spp. and *Photobacterium* spp. (*p* = 0.003). Spring and Autumn showed no statistical correlation among opportunistic pathogenic genera. In Hatchery A, an analysis revealed solely negative correlations among all studied genera across all seasons, although no analysis appeared statistically significant. Regarding Hatchery B, a positive statistically significant correlation was reported between *Vibrio* spp. and *Photobacterium* spp. (*p* = 0.000) in Winter samples. During Spring, similar to the HCMR hatchery, no statistically significant correlations were detected. Interestingly, during Autumn, all correlations appeared positive, with Vibrio spp. and *Photobacterium* spp. showing a statistically significant positive correlation (*p* = 0.000). These results suggest that the abundance of opportunistic fish pathogenic genera could be correlated in specific seasons.

### 3.5. Source Tracking of Putative Opportunistic Pathogens Among Sampling Points

**Figure 10:**
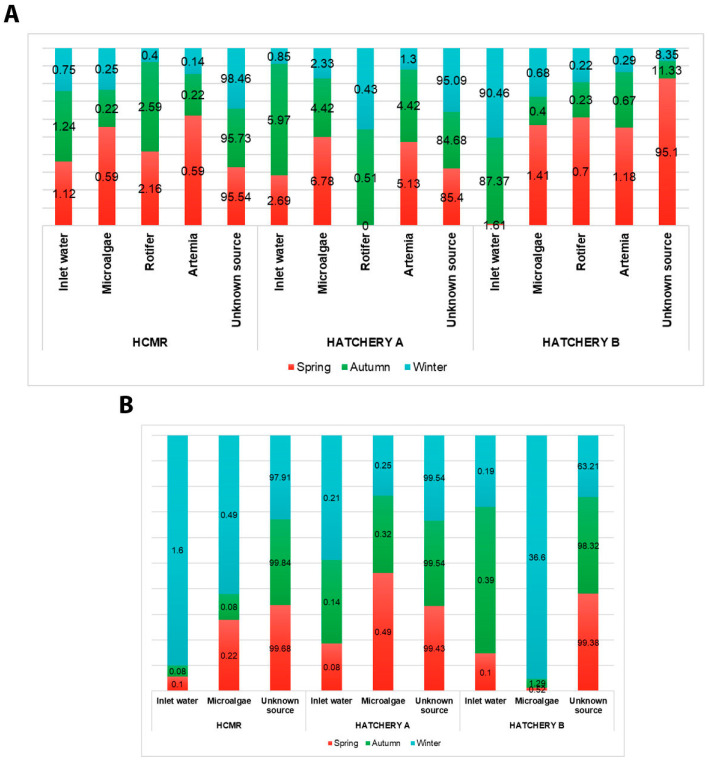
Source tracking of opportunistic fish pathogenic bacteria across seasons and hatcheries. The percentages of the potential sources of pathogen ASVs as derived by the SourceTracker analysis for every hatchery and season, separately. (**A**) Sources: Inlet water, microalgae, rotifers, Artemia, Sink: Outlet water; (**B**) Sources: Inlet water, microalgae, Sink: rotifer.

We also identified which sources were contributing the most to the abundance of the putative opportunistic fish pathogenic bacteria found in outlet water samples. Results presented in Figure 10A show that HCMR hatchery inlet water, rotifer, microalgae, and *Artemia* do not contribute significantly to the outlet water bacteriome regarding opportunistic fish pathogen ASVs. Only 4.46%, 4.27%, and 1.54% of the total opportunistic fish pathogen ASVs for Spring, Autumn, and Winter, respectively, were found to be sourced from the rest of the sampling points. On the other hand, these percentages are significant in Hatchery A, whereas 14.6%, 15.32%, and 4.91% of the total opportunistic fish pathogen ASVs for Spring, Autumn, and Winter, respectively, were found to be sourced by the rest of the sampling points. Interestingly, Hatchery B presented a greater increase, especially during Autumn and Winter, whereas the percentages were 4.9%, 88.6%, and 91.65% of the total opportunistic fish pathogen ASVs for Spring, Autumn, and Winter, respectively. More specifically, inlet water contributes the most, with 87.37% and 90.46% of the opportunistic fish pathogens being tracked during Autumn and Winter, respectively. These results demonstrate that the presence of opportunistic fish pathogens in the outlet water of live feeds is dependent on both the Hatchery and the season. Focusing on rotifers samples, we examined how much the inlet water and the microalgae contribute to the abundance of the opportunistic fish pathogenic bacteria described earlier. Results presented in Figure 10B show that in the HCMR hatchery, 0.32%, 0.16%, and 2.09% of inlet water contribute to the opportunistic fish pathogens of the rotifer during Spring, Autumn, and Winter, respectively. Similar results were recorded in Hatchery A, where only traces of opportunistic fish pathogens in rotifer samples were able to be tracked in inlet water and microalgae, with percentages being 0.57%, 0.46%, and 0.46% for Spring, Autumn, and Winter, respectively. A different picture was observed in Hatchery B during Winter, where 0.62%, 1.68%, and 36.79% of the total opportunistic fish pathogens were tracked in inlet water and microalgae during Spring, Autumn, and Winter, respectively. A total of 36.6% of the opportunistic fish pathogens during Winter were tracked from microalgae samples. We also identified which ASVs were the ones tracked from microalgae during Winter in Hatchery B. The results identified ASVs belonging to the *Vibrio* genus, and more specifically, they were ASV65, ASV355, ASV2271, ASV3220, ASV3220, ASV4418, ASV4545, ASV5346, ASV28, ASV5, ASV65, ASV355, ASV2271, ASV3220, ASV4418, ASV4545, ASV5346, ASV80, ASV170, ASV732, ASV3983, ASV38, ASV54, ASV55, ASV107, ASV168, ASV235, ASV271, ASV368, ASV426, ASV505, ASV563, ASV632, ASV819, ASV989, ASV992, ASV1006, ASV1446, ASV1560, ASV1647, ASV1887, ASV2914, ASV3007, ASV3343, ASV3503, ASV3568, ASV3765, ASV3789, ASV3829, ASV3838, ASV3889, ASV4159, ASV4600, ASV5050, ASV5422, ASV5899, ASV6271, ASV6511, ASV7040, ASV7042, ASV7202, ASV7765, ASV8248, ASV8250, and ASV50. Additionally, the results identified ASVs belonging to the *Tenacibaculum* genus, and more specifically, ASV20 and ASV7226. These results show that inlet water and microalgae could potentially shape the rotifer’s bacteriome communities under specific regimes.

## 4. Discussion

Mediterranean fish hatcheries face frequent bacterial outbreaks that are primarily caused by environmental endemic species [34]. As a result, commercial units invest resources in preventive measures to reduce the presence of fish pathogenic bacteria in their production line [34]. However, modern aquaculture systems rely on in-house systems to maintain or cultivate most of the live feeds used in the early stages of fish rearing, aiming for the development of a healthy fish gut bacteriome [35,36]. These systems can introduce endemic bacteria from the aquaculture environment as well as bacterial species from microalgae or zooplankton producers located far from Mediterranean ecosystems [7]. The high organic load that accumulates in microalgae live-feed production due to carbon fixation contributes significantly to bacterial growth and results in high bacterial titers [37]. Therefore, deep sequencing to characterize the microbiota of live feeds in Mediterranean aquaculture can offer valuable insights to update and improve rearing methodologies. To the best of our knowledge, only one recent work has been published involving Mediterranean fish hatcheries, describing their bacteriome profile by performing a deep sequencing analysis [38].

### 4.1. The Diversity of Microbiota of Fish Hatchery Live Feeds Is Distinctive and Depends on Multiple Factors

In our study, we examined three geographically distant Mediterranean closed fish hatchery systems with stable environmental conditions and found that although the reared fish species are typical of a Mediterranean aquaculture, the bacteriome structure can be unique and significantly divergent, and it is probably shaped by multiple industry-related factors, the inlet water, and the geographic location [38]. These features are likely to have a significant impact on the bacteriome of the produced juveniles, leading to differences in quality traits such as growth, survival, and robustness [39].

Several aspects of the analysis presented here lean towards a unique bacteriome structure developed in every hatchery. Although all three hatcheries are Mediterranean, a variation in terms of richness and diversity was observed from Chao1 and Shannon-index results, with Hatchery B (the only one using natural seawater) having a significantly richer and more diverse bacteriome structure when compared to the other two. Generally, environmental bacterial richness, especially from marine samples, can be predicted [40], but for samples taken from industrial cultures, such as live feeds, estimating any bacteria abundance is difficult. Typically, Mediterranean marine water Shannon index values of free-living bacteria have been reported above 3.9 [41,42], even reaching 6.1 [43], though in every analysis, we must consider the season, area, sequencing methodology and analysis, and other factors in order to compare diversity [44]. Nevertheless, in our case, we report relatively low Shannon diversity values in both genera and species levels, indicating an apparent lower bacterial diversity than would be typically expected in Mediterranean habitats. A major contributing factor to the reduced diversity observed is also the sterilization and filtration of the incoming water. All hatcheries, including the ones participating in our study, use UV sterilization to reduce the risk of bacterial contamination. It is obvious, however, that these systems cannot exclude the entrance of environmental bacteria into the hatchery system. On the other hand, they can reduce the overall number of bacterial titers and select bacterial species that are less sensitive to the sterilization process. This could be evidence of the selective enrichment of specific bacteria genera and species during live-feed cultivation, shifting the microbial diversity from a typical Mediterranean profile. Additionally, unique environmental conditions in every hatchery (Table 2), including the source of the inlet water (borehole water vs. natural seawater) and their geographic location, could potentially affect the bacteriome structure.

Geographic location has been proven to be an important factor when reporting microbiomes of fish larvae [45,46], but in the closed live-feed systems, its role remains unknown. The uniqueness of the bacteriome structures of fish hatcheries hints that their geographic location could have some effect [38]. It is also plausible to assume that the initial source of rotifer and *Artemia* batches, as well as microalgae inoculums, could be crucial factors for shaping a hatchery’s bacteriome diversity. In addition, the seasonal effect does not affect diversity greatly, as also previously concluded [38], and only in Hatchery B was a statistically significant increase observed during Spring compared to Winter and Autumn in both genus and ASV levels. Generally, seasonal effects and CO_2_ availability can greatly affect the Mediterranean’s bacteria diversity in the sea and natural environments [47,48], which is not the case in a controlled live-feed cultivation system, whereas conditions are optimized for juveniles’ healthcare and fitness.

### 4.2. Towards a Common Mediterranean Live-Feed Bacteriome

Some bacterial communities were consistently prevalent across different sampling points within the fish hatcheries studied, regardless of geographical location or season. Thus, Proteobacteria and Bacteroidetes were the most prevalent phyla in all three live-feed hatcheries studied, with Actinobacteria and Firmicutes contributing at a secondary level to the hatcheries’ diversity. *Alteromonas*, *Pseudoalteromonas*, *Nereida*, and *Phaeobacter* were the common genera among all three hatcheries studied. *Alteromonas* has been extensively studied due to the typing of deep-sea species of *Alteromonas macleodii* found in environmental bacteriome studies in the Mediterranean [49]. *Pseudoalteromonas* is abundant in the Mediterranean Sea [50], with some species, such as *Pseudoalteromonas marina*, being referred from Yellow Sea marine samples [51] and with *Pseudoalteromonas gelatinilytica* in samples in the East Pacific [52]. On the other hand, *Nereida* is a genus commonly found in Mediterranean marine waters, with its more common representative being *Nereida ignava*, cultivated and described in 2005 for the first time [47]. Finally, *Phaeobacter* is well-known by its representative species in the Mediterranean and North Sea, namely, *Phaeobacter portioli*, *Phaeobacter italicus*, and *Phaeobacter piscinae* [53,54,55], while other members of the genus, like *P. piscinae* and *P. inhibens*, display strong probiotic activities because they produce tropodithietic acid (TDA), a tropolone with antibacterial properties [56]. The presence of the *Phaeobacter* species within the live feed of fish hatcheries could contribute towards a microbial equilibrium, especially against opportunistic fish pathogenic genera. Interestingly, in vitro studies have highlighted the efficacy of *Phaeobacter* sp. against Vibrios [57,58]. The presence or incorporation of the *Phaeobacter* species within closed aquaculture systems could stabilize the seawater bacteriome [59]. Vibrios were found abundant in our core bacteriome analysis and prevalent as a common genus among all hatcheries. The *Vibrio* genus is represented by a large number of species in the Mediterranean Sea, posing a potential threat to aquaculture [60] despite the fact that they occur naturally in the gut of cultivated Mediterranean fish species [61]. Opportunistic pathogenic bacteria genera have also been reported as part of the core microbiota in other cases of Mediterranean hatcheries as well [38]. Reporting common bacteriomes among geographically distant hatcheries could set national-wide baseline information for improving hatchery management and, subsequently, fish health.

### 4.3. Putative Opportunistic Fish Pathogenic Genera Abundance Depends on Different Hatchery Strategies and Protocols

Three major bacterial genera known to include fish pathogens were detected in our samples. Several representative ASVs of the *Vibrio* genus were mainly detected in rotifers, microalgae, and *Artemia*. Although the 16s rDNA analysis on a species level is of low confidence, especially in genetically similar species such as *Vibrio*, our curated 16S database corresponded ASVs to some opportunistic important fish pathogenic Vibrio species, such as *Vibrio alginolyticus* and its genetically close relative, *Vibrio diabolicus* [62], which stand out as common fish pathogens among Mediterranean hatcheries and is one of the leading causative agents of vibriosis in Mediterranean aquaculture [63], with reported resistance to antibiotics [64] and implications as a zoonotic human pathogen through the food chain, also emerging partially due to climate change [65]. ASVs analysis also corresponded *Vibrio anguillarum* and *Vibrio harveyi*, which have exhibited a high potential risk against Mediterranean aquaculture [66,67]. Additionally, *Vibrio parahaemolyticus* is a human-related pathogen and a major concern in seafood safety [68]. Also, Splendidus clade within *Vibrio* is known to contain important pathogens for many aquatic animals, including fish [69]. Other possible species could be *Vibrio galatheae*, a recently described new member of the *Vibrionaceae* family, isolated initially during the Galathea 3 expedition in the Danish Sea from mussels [70], *Vibrio fortis*, a bacterium strongly related to dermatitis and enteric incidents in marine animals [71], and *Vibrio neptunis*, *Vibrio xuii*, and *Vibrio brasiliensis* species, which are highly associated with aquaculture live feeds, especially *Artemia* and rotifer [72]. Apart from opportunistic fish pathogenic *Vibrio* species, presumable oyster pathogenic bacteria could also be present in the HCMR hatchery, such as *Vibrio tubiashi* and *Vibrio coralliilyticus*, expanding the pathogenic capacity of the live feed beyond fish, concluding some of the *Vibrio* ASVs that corresponded to species as first result.

Apart from *Vibrio*, several species belonging to the *Tenacibaculum* and *Photobacterium* genera could emerge as important fish pathogens. Photobacteria are members of the *Vibrionacae* family, with two species being significant fish pathogens, including *Photobacterium damselae*, which has two subspecies, *P. damselae* subsp. piscicida, a causative agent of photobacteriosis (formerly known as pasteurellosis), and *P. damselae* subsp. *damselae* (formerly known as *Vibrio damsela*), an alternative causative agent of vibriosis. These two subspecies of *P. damselae*, although genetically close, have distinct phenotypes and can pose an immediate threat to marine animals [73]. Although, with ASV analysis, we could not distinguish between Photobacterium species or strains, in general, *Photobacterium* spp. was detected in all three hatcheries in relatively low abundance but in different seasons and sample sites, whereas HCMR had a higher abundance in inlet water during Spring.

In recent years, the emergence of specific *Tenacibaculum* species has posed a significant threat to aquacultures, which may also be influenced by climate change. Our analysis revealed that all three hatcheries showed a significant presence of *Tenacibaculum* spp., with ASVs corresponding to potent species such as *Tenacibaculum adriaticum*, *Tenacibaculum aestivarium*, and *Tenacibaculum aiptasiae* in their inlet and outlet water samples. The HCMR hatchery had a notably higher relative abundance of this genus in general despite it not being included in its core bacteriome. Moreover, *Tenacibaculum* spp., which could be identified either as *Tenacibaculum mesophilum* or *Tenacibaculum maritimum*, was found to be abundant in *Artemia* live feeds of Hatchery B, two closely genetically related putative fish pathogens [74].

Similarly to our *Vibrio* analysis, other possible *Tenacibaculum* species that our curated database showed were *Tenacibaculum adriaticum*, a newly described opportunistic fish pathogen [75], and *Tenacibaculum aestivarium*, which, although it has not yet been reported as a fish pathogen, Park et al. [76] identified it as a novel isolate from marine water, which, according to its genetically close relatives *Tenacibaculum dicentrarchi*, *Tenacibaculum soleae*, and *Tenacibaculum ovolyticum*, should be studied in detail as a potential threat for aquaculture.

However, the pathogenic potential of members of the *Tenacibaculum* genus is still under investigation due to significant difficulties in their isolation and identification, which limits the understanding of their actual role in clinical manifestations and hinders the routine fish diagnostic labs’ capabilities to identify them.

In the past, the co-occurrence of *Tenacibaculum* and *Vibrio* genera has been indicative of possible disease states in Atlantic Salmon hatcheries [77]; thus, studying their potential co-occurrence in bacteriome studies could be useful for preventing measures. Moreover, their correlation with seasonal variations could be useful for understanding when this co-occurrence is taking place with possible outbreak scenarios [78,79]. Herein, we identified a correlation abundance among the ASVs of three putative opportunistic pathogenic genera; namely, *Vibrio* spp., *Tenacibaculum* spp., and *Photobacterium* spp. Abundance correlation and co-occurrence of *Vibrio* and *Tenacibaculum* ASVs have been reported and linked with disease outbreaks in the aquaculture industry [77]. It is worth noting that during our monitoring program, no Vibriosis or Tenacibaculosis events were reported at the time samplings took place. Correlation analysis of opportunistic fish pathogenic abundance, either at the genera or ASVs level, could be utilized in the future as an important predictive tool.

Typically, the bacteriomes of aquaculture live-feed systems are highly regulated and controlled for the optimal growth and maintenance of live-feed organisms, and therefore, seasonality is not expected to significantly impact them [80,81,82]. However, changes in bacterial abundance are often observed, possibly due to the use of different water sources, the adoption of different batches of live feeds with varying qualities to meet industrial needs, seasonal preferences for marine animal rearing, and disinfection strategies for fish eggs, as also previously discussed and shown in Mediterranean hatcheries [38]. In our case, in regard to the HCMR hatchery (research-oriented hatchery), no disinfection of initial fish eggs took place, a strategy which could explain the high abundance of opportunistic fish pathogenic genera, which were not sourced from other sampling points, as seen in Figure 10 [83]. Additionally, our analysis of inlet water in Hatchery B revealed that during Winter and Autumn, it could be a source of opportunistic fish pathogenic genera, especially considering that this particular hatchery is using seawater (Figure 10; Table 2) compared to borehole water on the other hatcheries, which is known to pose some risks due to numerous factors [84].

Microalgae biomass can also contribute to the bacteriome structure of rotifers and could represent an important entry point for many bacterial species [85]. This is evident emphatically in the case of Hatchery B during Winter, as shown in Figure 10, while a significant contribution of inlet water and microalgae biomass in the observed rotifers’ bacteriome is also shown in other hatcheries as well. Nevertheless, it is already documented that rotifers’ and *Artemia* bacterial loads depend on the quality of the initial source [7,86] and can vary independently of the inlet water, the infrastructure, and the microalgae feed. Additionally, presence of Vibrios in live-feed cultures does not *de facto* mean that they will also appear in the outlet water, possibly due to antagonistic phenomena that have been well-described in eutrophic ecosystems among *Alteromonas*, *Pseudoalteromonas*, and *Vibrio* spp. [87,88,89,90,91]. It is, therefore, reasonable to hypothesize that similar antagonistic phenomena could occur in close eutrophic systems, such as live-feed aquaculture. This is supported by the presence of high abundances of the *Phaeobacter* genus, which are known to be antagonistic to Vibrios and were found in the studied hatcheries. The absence of disease outbreaks in the fish larval rearing tanks suggests a possible state of microbial equilibrium in all systems. However, putative fish pathogens could be present in the outlet water of all hatcheries, originating from inlet water, rotifers, *Artemia*, and microalgae in varying percentages of live feeds, which also may be part of a healthy bacteriome status rather than posing any direct risk of disease. This underscores the importance of monitoring potential Mediterranean fish pathogens throughout all stages of live-feed in-house cultivation pipelines.

## 5. Conclusions

Live feeds are critical for the health and resilience of fish larvae, and as such, they can significantly impact the quality and quantity of aquaculture production [92]. It has been shown that advanced molecular techniques can be instrumental in screening the bacteriome of large-scale live-feed cultures, enabling the identification of potential disease-related agents and the development of eco-friendly disinfection strategies [38]. Our research highlights the importance of studying the bacteriome of Mediterranean fish hatcheries, underlying that seasonality plays a secondary role. Furthermore, a diverse bacteriome can contain antagonistic organisms that inhibit the growth of fish pathogens, leading to better performance of fish larvae. This study presents a comprehensive analysis of the live-feed bacteriome of Mediterranean industrial hatcheries, utilizing the latest bioinformatics tools for future focus on well-known opportunistic fish pathogenic bacteria. Our findings provide valuable insights for promoting a more sustainable and less drug-dependent aquaculture sector.

## Figures and Tables

**Table 1:** Environmental conditions of the 5 sampling stations in the present study.

	Inlet Water			Rotifer			*Artemia*			Microalgae			Outlet Water		
	pH	S (‰)	T (°C)	pH	S (‰)	T (°C)	pH	S (‰)	T (°C)	pH	S (‰)	T (°C)	pH	S (‰)	T (°C)
HCMR hatchery	7.4	34	19	7.5	25	25	7.3–7.8	25–28	25	7.5	34	26.5–29	7.5	34	19–20
Hatchery A	7.1	36	19	7.8–8.5	36	20	7.5	25	25	7.5	36	25	7	36	19
Hatchery B	7.9–8.2	40	14–28	7.8–8.4	40	22–24	7.6	20	26–28	7.5	40	28	7.7–8	40	14–28

**Table 2:** Main attributes of the hatcheries used in the present study.

Hatchery Name	Live Feed Water Supply	Cultivated Microalgae Species	Type of Hatchery	Main Cultivated Fish Species
HCMR hatchery	Borehole water	*Chlorella minutissima*	Research	Gilthead seabream, European seabass, meagre, greater amberjack
Hatchery A	Borehole water	*Isochrisis* sp.	Commercial	Gilthead seabream, European seabass
Hatchery B	Sea Water	*Tetraselmis* sp.	Commercial	Gilthead seabream, European seabass

**Table 3:** Core bacterial structure of all the hatcheries based on common genera among all sampling points and during all three studied seasons (N/A for not available).

Phylum	Class	Order	Family	Genus	
Proteobacteria	Gammaproteobacteria	Alteromonadales	*Pseudoalteromonadaceae*	*Pseudoalteromonas*	HCMR HATCHERY
Proteobacteria	Gammaproteobacteria	Alteromonadales	*Alteromonadaceae*	*Alteromonas*	
Bacteroidetes	Flavobacteriia	Flavobacteriales	*Flavobacteriaceae*	*Olleya*	
Actinobacteria	Actinomycetia	Micrococcales	*Micrococcaceae*	*Glutamicibacter*	
Proteobacteria	Gammaproteobacteria	Alteromonadales	*Alteromonadaceae*	*Marisediminitalea*	
Proteobacteria	Alphaproteobacteria	Rhodobacterales	*Rhodobacteraceae*	*Tritonibacter*	
Proteobacteria	Alphaproteobacteria	Rhodobacterales	*Roseobacteraceae*	*Nereida*	
Proteobacteria	Alphaproteobacteria	Rhodobacterales	*Roseobacteraceae*	*Phaeobacter*	
Proteobacteria	Gammaproteobacteria	Vibrionales	*Vibrionaceae*	*Vibrio*	
Bacteroidetes	Bacteroidia	Flavobacteriales	*Flavobacteriaceae*	N/A	HATCHERY A
Bacteroidetes	Bacteroidia	Marinilabiliales	*Prolixibacteraceae*	*Sunxiuqinia*	
Proteobacteria	Alphaproteobacteria	Rhodobacterales	*Roseobacteraceae*	*Phaeobacter*	
Proteobacteria	Gammaproteobacteria	Vibrionales	*Vibrionaceae*	*Vibrio*	
Proteobacteria	Alphaproteobacteria	Rhodobacterales	*Roseobacteraceae*	*Roseovarius*	
Proteobacteria	Gammaproteobacteria	Alteromonadales	*Alteromonadaceae*	*Alteromonas*	
Proteobacteria	Alphaproteobacteria	Rhodobacterales	*Roseobacteraceae*	*Nereida*	
Proteobacteria	Alphaproteobacteria	Rhodobacterales	*Roseobacteraceae*	*Roseovarius*	
Patescibacteria	Gracilibacteria	JGI_0000069-P22	*JGI_0000069-P22*	*JGI 0000069-P22*	
Bacteroidetes	Bacteroidia	Flavobacteriales	*Flavobacteriaceae*	N/A	
Proteobacteria	Gammaproteobacteria	Alteromonadales	*Pseudoalteromonadaceae*	*Pseudoalteromonas*	
Proteobacteria	Gammaproteobacteria	Alteromonadales	*Idiomarinaceae*	*Pseudidiomarina*	
Proteobacteria	Gammaproteobacteria	Vibrionales	*Vibrionaceae*	*Catenococcus*	HATCHERY B
Proteobacteria	Gammaproteobacteria	Vibrionales	*Vibrionaceae*	*Vibrio*	
Proteobacteria	Alphaproteobacteria	Rhodobacterales	*Roseobacteraceae*	*Nereida*	
Proteobacteria	Gammaproteobacteria	Alteromonadales	*Alteromonadaceae*	*Alteromonas*	
Bacteroidetes	Flavobacteriia	Flavobacteriales	*Flavobacteriaceae*	*Maribacter*	
Proteobacteria	Alphaproteobacteria	Rhodobacterales	*Rhodobacteraceae*	*Tritonibacter*	
Proteobacteria	Gammaproteobacteria	Alteromonadales	*Pseudoalteromonadaceae*	*Pseudoalteromonas*	
Bacteroidetes	Flavobacteriia	Flavobacteriales	*Flavobacteriaceae*	*Tenacibaculum*	
Proteobacteria	Alphaproteobacteria	Rhodobacterales	*Roseobacteraceae*	*Phaeobacter*	
Proteobacteria	Alphaproteobacteria	Rhodobacterales	*Roseobacteraceae*	*Roseovarius*	
Proteobacteria	Alphaproteobacteria	Rhodobacterales	*Roseobacteraceae*	*Donghicola*	
Proteobacteria	Gammaproteobacteria	Oceanospirillales	*Oceanospirillaceae*	*Marinomonas*	
Proteobacteria	Gammaproteobacteria	Alteromonadales	*Alteromonadaceae*	*Alteromonas*	

## Data Availability

All the raw sequence files (corresponding information at Supplemental Meta Data of Metagenomic Libraries) of this study were submitted to the European Nucleotide Archive (ENA) [93] with the study accession number PRJEB59727 and are available at http://www.ebi.ac.uk/ena/data/view/PRJEB59727, accessed on 23 March 2023. All data are available upon request to the authors.

## Supplementary Materials

The following supporting information can be downloaded at https://www.mdpi.com/article/10.3390/microorganisms12112281/s1, Figure S1. Schematic representation of the sampling stations, the number of samples per season and per hatchery, as well as how inlet water, live feeds (microalgae, rotifers and Artemia) and outlet water interact with of each other including the fish larval rearing tanks in all three hatcheries studied. Figure S2. Rarefaction curves of the annotated sequences generated from Novaseq sequencing corresponding in genera and ASVs level. Figure S3. Principal component analysis (PCA) of genera abundance in live feeds of (A) HCMR hatchery, (B) Hatchery A, (C) Hatchery B. Figure S4. Loadings of PCAs from Figure S3, genera relative abundances in live feeds of (A) HCMR hatchery, (B) Hatchery A, (C) Hatchery B. Figure S5. Phyla of most prevalent bacteria in (A) HCMR hatchery, (B) Hatchery A and (C) Hatchery B. Supplementary Data. Average Relative abundances of most abundant genera in all hatcheries. Supplemental Meta Data of Metagenomic Libraries. Information regarding the raw sequence files of this study, which are submitted to the European Nucleotide Archive (ENA). Table S1. Grand Mean and Standard Error of Means (SEM) of relative abundances of the presumably opportunistic fish pathogenic species after our ASV blast analysis (first result) in HCMR hatchery among (a) sampling point and (b) among seasons. Different superscript letters indicate where the statistically significant difference occurs (One-way ANOVA, *p* ≤ 0.05, Post-hoc test LSD; n/a for not available). Table S2. Grand Mean and Standard Error of Means (SEM) of relative abundances of the presumably opportunistic fish pathogenic species after our ASV blast analysis (first result) in Hatchery A among (a) sampling point and (b) among seasons. Table S3. Grand Mean and Standard Error of Means (SEM) of relative abundances of the presumably opportunistic fish pathogenic species after our ASV blast analysis (first result) in Hatchery B among (a) sampling point and (b) among seasons.

## References

[1] Almeida D.B., Magalhães C., Sousa Z., Borges M.T., Silva E., Blanquet I., Mucha A.P. (2021). Microbial community dynamics in a hatchery recirculating aquaculture system (RAS) of sole (*Solea senegalensis*). Aquaculture.

[2] Bugten A.V., Attramadal K.J.K., Fossmark R.O., Rosten T.W., Vadstein O., Bakke I. (2022). Changes in rearing water microbiomes in RAS induced by membrane filtration alters the hindgut microbiomes of Atlantic salmon (*Salmo salar*) parr. Aquaculture.

[3] Lillehaug A., Santi N., Østvik A. (2015). Practical Biosecurity in Atlantic Salmon Production. J. Appl. Aquac..

[4] Padrós F., Caggiano M., Toffan A., Constenla M., Zarza C., Ciulli S. (2022). Integrated Management Strategies for Viral Nervous Necrosis (VNN) Disease Control in Marine Fish Farming in the Mediterranean. Pathogens.

[5] Stankus A. (2021). State of world aquaculture 2020 and regional reviews: FAO webinar series. FAO Aquac. Newsl..

[6] Zapata A., Diez B., Cejalvo T., Frías C.G.-D., Cortés A. (2006). Ontogeny of the immune system of fish. Fish Shellfish Immunol..

[7] Conceição L.E., Yúfera M., Makridis P., Morais S., Dinis M.T. (2010). Live feeds for early stages of fish rearing. Aquac. Res..

[8] Paulo M.C., Cardoso C., Coutinho J., Castanho S., Bandarra N.M. (2020). Microalgal solutions in the cultivation of rotifers and artemia: Scope for the modulation of the fatty acid profile. Heliyon.

[9] Nakase G., Nakagawa Y., Miyashita S., Nasu T., Senoo S., Matsubara H., Eguchi M. (2007). Association between bacterial community structures and mortality of fish larvae in intensive rearing systems. Fish. Sci..

[10] Infante-Villamil S., Huerlimann R., Jerry D.R. (2021). Microbiome diversity and dysbiosis in aquaculture. Rev. Aquac..

[11] Bentzon-Tilia M., Sonnenschein E.C., Gram L. (2016). Monitoring and managing microbes in aquaculture–Towards a sustainable industry. Microb. Biotechnol..

[12] Llewellyn M.S., Boutin S., Hoseinifar S.H., Derome N. (2014). Teleost microbiomes: The state of the art in their characterization, manipulation and importance in aquaculture and fisheries. Front. Microbiol..

[13] Romero J., Navarrete P. (2006). 16S rDNA-based analysis of dominant bacterial populations associated with early life stages of coho salmon (*Oncorhynchus kisutch*). Microb. Ecol..

[14] Dittmann K.K., Rasmussen B.B., Melchiorsen J., Sonnenschein E.C., Gram L., Bentzon-Tilia M. (2020). Changes in the microbiome of mariculture feed organisms after treatment with a potentially probiotic strain of *Phaeobacter inhibens*. Appl. Environ. Microbiol..

[15] Wikfors G.H., Ohno M. (2001). Impact of algal research in aquaculture. J. Phycol..

[16] Uren Webster T.M., Rodriguez-Barreto D., Castaldo G., Gough P., Consuegra S., Garcia de Leaniz C. (2020). Environmental plasticity and colonization history in the Atlantic salmon microbiome: A translocation experiment. Mol. Ecol..

[17] Odom A.R., Faits T., Castro-Nallar E., Crandall K.A., Johnson W.E. (2023). Metagenomic profiling pipelines improve taxonomic classification for 16S amplicon sequencing data. Sci. Rep..

[18] Levican A., Fisher J.C., McLellan S.L., Avendaño-Herrera R. (2020). Microbial communities associated with farmed Genypterus chilensis: Detection in water prior to bacterial outbreaks using culturing high-throughput sequencing. Animals.

[19] Goldberg C.S., Turner C.R., Deiner K., Klymus K.E., Thomsen P.F., Murphy M.A., Spear S.F., McKee A., Oyler-McCance S.J., Cornman R.S. (2016). Critical considerations for the application of environmental DNA methods to detect aquatic species. Methods Ecol. Evol..

[20] Cowart D.A., Murphy K.R., Cheng C.H.C. (2018). Metagenomic sequencing of environmental DNA reveals marine faunal assemblages from the West Antarctic Peninsula. Mar. Genom..

[21] Natarajan V.P., Zhang X., Morono Y., Inagaki F., Wang F. (2016). A modified SDS-based DNA extraction method for high quality environmental DNA from seafloor environments. Front. Microbiol..

[22] Klindworth A., Pruesse E., Schweer T., Peplies J., Quast C., Horn M., Glöckner F.O. (2013). Evaluation of general 16S ribosomal RNA gene PCR primers for classical and next-generation sequencing-based diversity studies. Nucleic Acids Res..

[23] Bolyen E., Rideout J.R., Dillon M.R., Bokulich N.A., Abnet C.C., Al-Ghalith G.A., Alexander H., Alm E.J., Arumugam M., Asnicar F. (2019). Reproducible, interactive, scalable and extensible microbiome data science using QIIME 2. Nat. Biotechnol..

[24] Callahan B.J., McMurdie P.J., Rosen M.J., Han A.W., Johnson A.J.A., Holmes S.P. (2016). DADA2: High-resolution sample inference from Illumina amplicon data. Nat. Methods.

[25] Callahan B.J., McMurdie P.J., Holmes S.P. (2017). Exact sequence variants should replace operational taxonomic units in marker-gene data analysis. ISME J..

[26] Altschul S.F., Gish W., Miller W., Myers E.W., Lipman D.J. (1990). Basic local alignment search tool. J. Mol. Biol..

[27] Bokulich N.A., Kaehler B.D., Rideout J.R., Dillon M., Bolyen E., Knight R., Huttley G.A., Gregory Caporaso J. (2018). Optimizing taxonomic classification of marker-gene amplicon sequences with QIIME 2’s q2-feature-classifier plugin. Microbiome.

[28] Weiss S., Xu Z.Z., Peddada S., Amir A., Bittinger K., Gonzalez A., Lozupone C., Zaneveld J.R., Vázquez-Baeza Y., Birmingham A. (2017). Normalization and microbial differential abundance strategies depend upon data characteristics. Microbiome.

[29] McMurdie J.P., Holmes S. (2013). phyloseq: An R package for reproducible interactive analysis and graphics of microbiome census data. PLoS ONE..

[30] Oksanen J., Blanchet F.G., Friendly M., Kindt R., Legendre P., McGlinn D., Minchin P.R., O’Hara R.B., Simpson G.L., Solymos P. vegan: Community Ecology Package. R Package Version 2.5-4. 2019. Computer Software. https://cran.r-project.org/web/packages/vegan/index.html.

[31] Knights D., Kuczynski J., Charlson E.S., Zaneveld J., Mozer M.C., Collman R.G., Bushman F.D., Knight R., Kelley S.T. (2011). Bayesian community-wide culture-independent microbial source tracking. Nat. Methods.

[32] Tackmann J., Rodrigues J.F.M., von Mering C. (2019). Rapid inference of direct interactions in large-scale ecological networks from heterogeneous microbial sequencing data. Cell Syst..

[33] Shetty S.A., Lahti L. (2019). Microbiome data science. J. Biosci..

[34] Vendramin N., Zrncic S., Padrós F., Oraic D., Le Breton A., Zarza C., Olesen N.J. (2016). Fish health in Mediterranean Aquaculture, past mistakes and future challenges. Bull. Eur. Assoc. Fish Pathol..

[35] Ringø E., Olsen R.E. (1999). The effect of diet on aerobic bacterial flora associated with intestine of Arctic charr (*Salvelinus alpinus L.*). J. Appl. Microbiol..

[36] Egerton S., Culloty S., Whooley J., Stanton C., Ross R.P. (2018). The gut microbiota of marine fish. Front. Microbiol..

[37] Salvesen I., Reitan K.I., Skjermo J., Òie G. (2000). Microbial environments in marine larviculture: Impacts of algal growth rates on the bacterial load in six microalgae. Aquac. Int..

[38] Najafpour B., Pinto P.I., Sanz E.C., Martinez-Blanch J.F., Canario A.V., Moutou K.A., Power D.M. (2023). Core microbiome profiles and their modification by environmental, biological, and rearing factors in aquaculture hatcheries. Mar. Pollut. Bull..

[39] Almeida D.B., Semedo M., Magalhães C., Blanquet I., Mucha A.P. (2023). Sole microbiome progression in a hatchery life cycle, from egg to juvenile. Front. Microbiol..

[40] Hong S.H., Bunge J., Jeon S.O., Epstein S.S. (2006). Predicting microbial species richness. Proc. Natl. Acad. Sci. USA.

[41] Pommier T., Neal P.R., Gasol J.M., Coll M., Acinas S.G., Pedrós-Alió C. (2010). Spatial patterns of bacterial richness and evenness in the NW Mediterranean Sea explored by pyrosequencing of the 16S rRNA. Aquat. Microb. Ecol..

[42] Kerfahi D., Hall-Spencer J.M., Tripathi B.M., Milazzo M., Lee J., Adams J.M. (2014). Shallow water marine sediment bacterial community shifts along a natural CO_2_ gradient in the Mediterranean Sea off Vulcano, Italy. Microb. Ecol..

[43] Pop Ristova P., Wenzhöfer F., Ramette A., Felden J., Boetius A. (2015). Spatial scales of bacterial community diversity at cold seeps (Eastern Mediterranean Sea). ISME J..

[44] Luna G.M. (2015). Diversity of marine microbes in a changing Mediterranean Sea. Rend. Lincei.

[45] Bakke I., Coward E., Andersen T., Vadstein O. (2015). Selection in the host structures the microbiota associated with developing cod larvae (*Gadus morhua*). Environ. Microbiol..

[46] Califano G., Castanho S., Soares F., Ribeiro L., Cox C.J., Mata L., Costa R. (2017). Molecular taxonomic profiling of bacterial communities in a gilthead seabream (*Sparus aurata*) hatchery. Front. Microbiol..

[47] Mestre M., Höfer J., Sala M.M., Gasol J.M. (2020). Seasonal variation of bacterial diversity along the marine particulate matter continuum. Front. Microbiol..

[48] López-Pérez M., Gonzaga A., Martin-Cuadrado A.B., Onyshchenko O., Ghavidel A., Ghai R., Rodriguez-Valera F. (2012). Genomes of surface isolates of *Alteromonas macleodii*: The life of a widespread marine opportunistic copiotroph. Sci. Rep..

[49] Xu F., Cha Q.Q., Zhang Y.Z., Chen X.L. (2021). Degradation and utilization of alginate by marine *Pseudoalteromonas*: A Review. Appl. Environ. Microbiol..

[50] Nam Y.D., Chang H.W., Park J.R., Kwon H.Y., Quan Z.X., Park Y.H., Lee J.S., Yoon J.H., Bae J.W. (2007). *Pseudoalteromonas marina* sp. nov., a marine bacterium isolated from tidal flats of the Yellow Sea, and reclassification of *Pseudoalteromonas sagamiensis* as *Algicola sagamiensis* comb. nov. Int. J. Syst. Evol. Microbiol..

[51] Yan J., Wu Y.H., Meng F.X., Wang C.S., Xiong S.L., Zhang X.Y., Zhang Y.Z., Xu X.W., Zhang D.M. (2016). *Pseudoalteromonas gelatinilytica* sp. nov., isolated from surface seawater. Int. J. Syst. Evol. Microbiol..

[52] Pujalte M.J., Macián M.C., Arahal D.R., Ludwig W., Schleifer K.H., Garay E. (2005). *Nereida ignava* gen. nov., sp. nov., a novel aerobic marine α-proteobacterium that is closely related to uncultured *Prionitis* (alga) gall symbionts. Int. J. Syst. Evol. Microbiol..

[53] Sonnenschein E.C., Jimenez G., Castex M., Gram L. (2021). The Roseobacter-group bacterium *Phaeobacter* as a safe probiotic solution for aquaculture. Appl. Environ. Microbiol..

[54] Breider S., Freese H.M., Spröer C., Simon M., Overmann J., Brinkhoff T. (2017). *Phaeobacter porticola* sp. nov., an antibiotic-producing bacterium isolated from a sea harbour. Int. J. Syst. Evol. Microbiol..

[55] Sonnenschein E.C., Phippen C.B.W., Nielsen K.F., Mateiu R.V., Melchiorsen J., Gram L., Overmann J., Freese H.M. (2017). *Phaeobacter piscinae* sp. nov., a species of the Roseobacter group and potential aquaculture probiont. Int. J. Syst. Evol. Microbiol..

[56] Henriksen N.N., Lindqvist L.L., Wibowo M., Sonnenschein E.C., Bentzon-Tilia M., Gram L. (2022). Role is in the eye of the beholder—The multiple functions of the antibacterial compound tropodithietic acid produced by marine *Rhodobacteraceae*. FEMS Microbiol. Rev..

[57] D’Alvise P.W., Lillebø S., Prol-Garcia M.J., Wergeland H.I., Nielsen K.F., Bergh Ø., Gram L. (2012). *Phaeobacter gallaeciensis* reduces *Vibrio anguillarum* in cultures of microalgae and rotifers, and prevents vibriosis in cod larvae. PLoS ONE.

[58] Roager L., Athena-Vasileiadi D., Gram L., Sonnenschein E.C. (2024). Antagonistic activity of *Phaeobacter piscinae* against the emerging fish pathogen *Vibrio crassostreae* in aquaculture feed algae. Appl. Environ. Microbiol..

[59] Prol-García M.J., Gómez M., Sánchez L., Pintado J. (2014). *Phaeobacter* grown in biofilters: A new strategy for the control of *Vibrionaceae* in aquaculture. Aquac. Res..

[60] Colwell R.R., Grimes D.J. (1984). Vibrio diseases of marine fish populations. Helgoländer Meeresunters..

[61] Onarheim A.M., Wiik R., Burghardt J., Stackebrandt E. (1994). Characterization and identification of two Vibrio species indigenous to the intestine of fish in cold sea water; description of *Vibrio iliopiscarius* sp. nov. Syst. Appl. Microbiol..

[62] Raguénès G., Christen R., Guezennec J., Pignet P., Barbier G. (1997). *Vibrio diabolicus* sp. nov., a new polysaccharide-secreting organism isolated from a deep-sea hydrothermal vent polychaete annelid, *Alvinella pompejana*. Int. J. Syst. Evol. Microbiol..

[63] Zhao Z., Chen C., Hu C.Q., Ren C.H., Zhao J.J., Zhang L.P., Jiang X., Luo P., Wang Q.B. (2010). The type III secretion system of *Vibrio alginolyticus* induces rapid apoptosis, cell rounding and osmotic lysis of fish cells. Microbiology.

[64] Oh E.G., Son K.T., Yu H., Lee T.S., Lee H.J., Shin S., Kwon J.Y., Park K., Kim J. (2011). Antimicrobial resistance of *Vibrio parahaemolyticus* and *Vibrio alginolyticus* strains isolated from farmed fish in Korea from 2005 through 2007. J. Food Prot..

[65] Mustapha S., Mustapha E.M., Nozha C. (2013). *Vibrio alginolyticus*: An emerging pathogen of foodborne diseases. Int. J. Sci. Technol..

[66] Austin B., Austin D.A. (2007). Characteristics of the pathogens: Gram-negative bacteria. Bacterial Fish Pathogens: Diseases of Farmed and Wild Fish.

[67] Triga A., Smyrli M., Katharios P. (2023). Pathogenic and opportunistic *Vibrio* spp. associated with vibriosis incidences in the Greek aquaculture: The role of *Vibrio harveyi* as the principal cause of vibriosis. Microorganisms.

[68] Su Y.C., Liu C. (2007). *Vibrio parahaemolyticus*: A concern of seafood safety. Food Microbiol..

[69] Beaz-Hidalgo R., Diéguez A.L., Cleenwerck I., Balboa S., Doce A., De Vos P., Romalde J.L. (2010). *Vibrio celticus* sp. nov., a new Vibrio species belonging to the Splendidus clade with pathogenic potential for clams. Syst. Appl. Microbiol..

[70] Giubergia S., Machado H., Mateiu R.V., Gram L. (2016). Vibrio galatheae sp. nov., a member of the family *Vibrionaceae* isolated from a mussel. Int. J. Syst. Evol. Microbiol..

[71] Evans D., Millar Z., Harding D., Pham P.H., LePage V., Lumsden J.S. (2022). Lipoid liver disease in Hippocampus erectus Perry with *Vibrio fortis*-induced dermatitis and enteritis. J. Fish Dis..

[72] Thompson F.L., Li Y., Gomez-Gil B., Thompson C.C., Hoste B., Vandemeulebroecke K., Rupp G.S., Pereira A., De Bem M.M., Sorgeloos P. (2003). *Vibrio neptunius* sp. nov., *Vibrio brasiliensis* sp. nov. and *Vibrio xuii* sp. nov., isolated from the marine aquaculture environment (bivalves, fish, rotifers and shrimps). Int. J. Syst. Evol. Microbiol..

[73] Rivas A.J., Lemos M.L., Osorio C.R. (2013). *Photobacterium damselae* subsp. damselae, a bacterium pathogenic for marine animals and humans. Front. Microbiol..

[74] Miyake S., Soh M., Ding Y., Seedorf H. (2019). Complete genome sequence of sponge-associated *Tenacibaculum mesophilum* DSM 13764T. Microbiol. Resour. Announc..

[75] Heindl H., Wiese J., Imhoff J.F. (2008). *Tenacibaculum adriaticum* sp. nov., from a bryozoan in the Adriatic Sea. Int. J. Syst. Evol. Microbiol..

[76] Park S., Choi S.J., Won S.M., Yoon J.H. (2017). *Tenacibaculum aestuariivivum* sp. nov., isolated from a tidal flat. Int. J. Syst. Evol. Microbiol..

[77] Wynne J.W., Thakur K.K., Slinger J., Samsing F., Milligan B., Powell J.F., McKinnon A., Nekouei O., New D., Richmond Z. (2020). Microbiome profiling reveals a microbial dysbiosis during a natural outbreak of tenacibaculosis (Yellow mouth) in Atlantic salmon. Front. Microbiol..

[78] Debroas D., Hochart C., Galand P.E. (2022). Seasonal microbial dynamics in the ocean inferred from assembled and unassembled data: A view on the unknown biosphere. ISME Commun..

[79] Kobiyama A., Rashid J., Reza M.S., Ikeda Y., Yamada Y., Kudo T., Mizusawa N., Yanagisawa S., Ikeda D., Sato S. (2021). Seasonal and annual changes in the microbial communities of Ofunato Bay, Japan, based on metagenomics. Sci. Rep..

[80] Kumar G.R., Babu D.E. (2015). Effect of light, temperature and salinity on the growth of *Artemia*. Int. J. Eng. Sci. Invent..

[81] Lee M.C., Yoon D.S., Park J.C., Choi H., Shin K.H., Hagiwara A., Lee J.S., Park H.G. (2022). Effects of salinity and temperature on reproductivity and fatty acid synthesis in the marine rotifer *Brachionus rotundiformis*. Aquaculture.

[82] Pan Y., Dahms H., Hwang J., Souissi S. (2022). Recent Trends in Live Feeds for Marine Larviculture: A Mini Review. Front. Mar. Sci..

[83] Lahnsteiner F. (2021). Effect of disinfection of non-hardened Salmo trutta eggs with Chloramine T^®^, Wofasteril^®^, and hydrogen peroxide on embryo and larvae viability, microorganism load, lipid peroxidation, and protein carbonylation. Aquac. Int..

[84] Elgendy M.Y., Ali S.E., Abbas W.T., Algammal A.M., Abdelsalam M. (2023). The role of marine pollution on the emergence of fish bacterial diseases. Chemosphere.

[85] Natrah F.M., Bossier P., Sorgeloos P., Yusoff F.M., Defoirdt T. (2014). Significance of microalgal–bacterial interactions for aquaculture. Rev. Aquac..

[86] Dhont J., Dierckens K., Støttrup J., Van Stappen G., Wille M., Sorgeloos P. (2013). Rotifers, *Artemia* and copepods as live feeds for fish larvae in aquaculture. Advances in Aquaculture Hatchery Technology.

[87] Gram L., Melchiorsen J., Spanggaard B., Huber I., Nielsen T.F. (1999). Inhibition of *Vibrio anguillarum* by *Pseudomonas fluorescens* AH2, a possible probiotic treatment of fish. Appl. Environ. Microbiol..

[88] Long R.A., Rowley D.C., Zamora E., Liu J., Bartlett D.H., Azam F. (2005). Antagonistic interactions among marine bacteria impede the proliferation of *Vibrio cholerae*. Appl. Environ. Microbiol..

[89] Del Castillo C.S., Wahid M.I., Yoshikawa T., Sakaia T. (2008). Isolation and inhibitory effect of anti-*Vibrio* substances from *Pseudoalteromonas* sp. A1-J11 isolated from the coastal sea water of Kagoshima Bay. Fish. Sci..

[90] Morya V.K., Choi W., Kim E.K. (2014). Isolation and characterization of *Pseudoalteromonas* sp. from fermented Korean food, as an antagonist to *Vibrio harveyi*. Appl. Microbiol. Biotechnol..

[91] Shen H., Song T., Lu J., Qiu Q., Chen J., Xiong J. (2021). Shrimp AHPND causing *Vibrio anguillarum* infection: Quantitative diagnosis and identifying antagonistic bacteria. Mar. Biotechnol..

[92] Hansen G.H., Olafsen J.A. (1999). Bacterial interactions in early life stages of marine cold water fish. Microb. Ecol..

[93] Burgin J., Ahamed A., Cummins C., Devraj R., Gueye K., Gupta D., Gupta V., Haseeb M., Ihsan M., Ivanov E. (2023). The European Nucleotide Archive in 2022. Nucleic Acids Res..

