# Supplementary material for: Unveiling Emerging Opportunistic Fish Pathogens in Aquaculture: A Comprehensive Seasonal Study of Microbial Composition in Mediterranean Fish Hatcheries
Source: Microorganisms. 2024 Nov 10;12(11):2281. doi: 10.3390/microorganisms12112281 (PMC11596916; doi:10.3390/microorganisms12112281)
Supplement: Supplementary file 1 [file microorganisms-12-02281-s001.zip › Supplemental Figures.pdf]

Unveiling emerging opportunistic fish pathogens in aquaculture: A comprehensive seasonal study of microbial composition in Mediterranean fish hatcheries.

Dimitrios Skliros[1], Maria Kostakou[1,2], Constantina Kokkari[3], Maria Ioanna Tsertou[3], Christina Pavloudi[4], Haris Zafeiropoulos[5], Pantelis Katharios[3], Emmanouil Flemetakis[1,]*

[1]Laboratory of Molecular Biology, Department of Biotechnology, School of Applied Biology and Biotechnology, Agricultural University of Athens, 1855 Athens, Greece

[2]Department of Applied Microbial Ecology, Helmholtz Centre for Environmental Research- UFZ, Leipzig, 4318, Leipzig, Germany

[3]Institute of Marine Biology, Biotechnology and Aquaculture, Hellenic Centre for Marine Research, 71500 Heraklion, Greece

[4]European Marine Biological Resource Centre-European Research Infrastructure Consortium (EMBRC-ERIC), 75252 Paris, France

[5]Laboratory of Molecular Bacteriology, Rega Institute for Medical Research, Department of Microbiology, Immunology and Transplantation, KU Leuven, 3000 Leuven, Belgium

*Author to whom correspondence should be addressed: Emmanouil Flemetakis, Laboratory of Molecular Biology, Department of Biotechnology, School of Applied Biology and Biotechnology, Agricultural University of Athens, 11855 Athens, Greece, mflem@aua.gr, 00302105294343

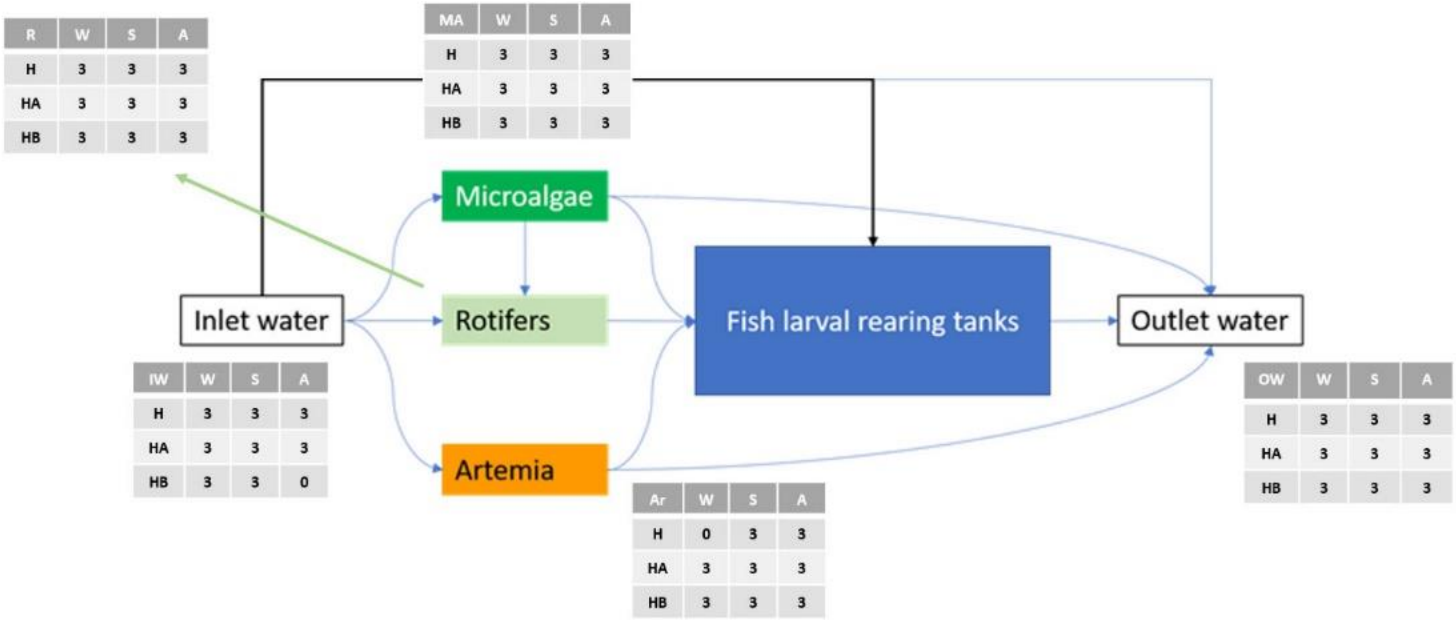

Figure S1. Schematic representation of the sampling stations, the number of samples per season and per hatchery, as well as how inlet water, live feeds (microalgae, rotifers and *Artemia*) and outlet water interact with of each other including the fish larval rearing tanks in all three hatcheries studied. W→Winter, S→Spring, A→Autumn, H→HCMR hatchery, HA→Hatchery A, HB→ Hatchery B, R→ Rotifer, IW→ Inlet Water, OW→Outlet Water, Ar→ Artemia, MA→ Microalgae.

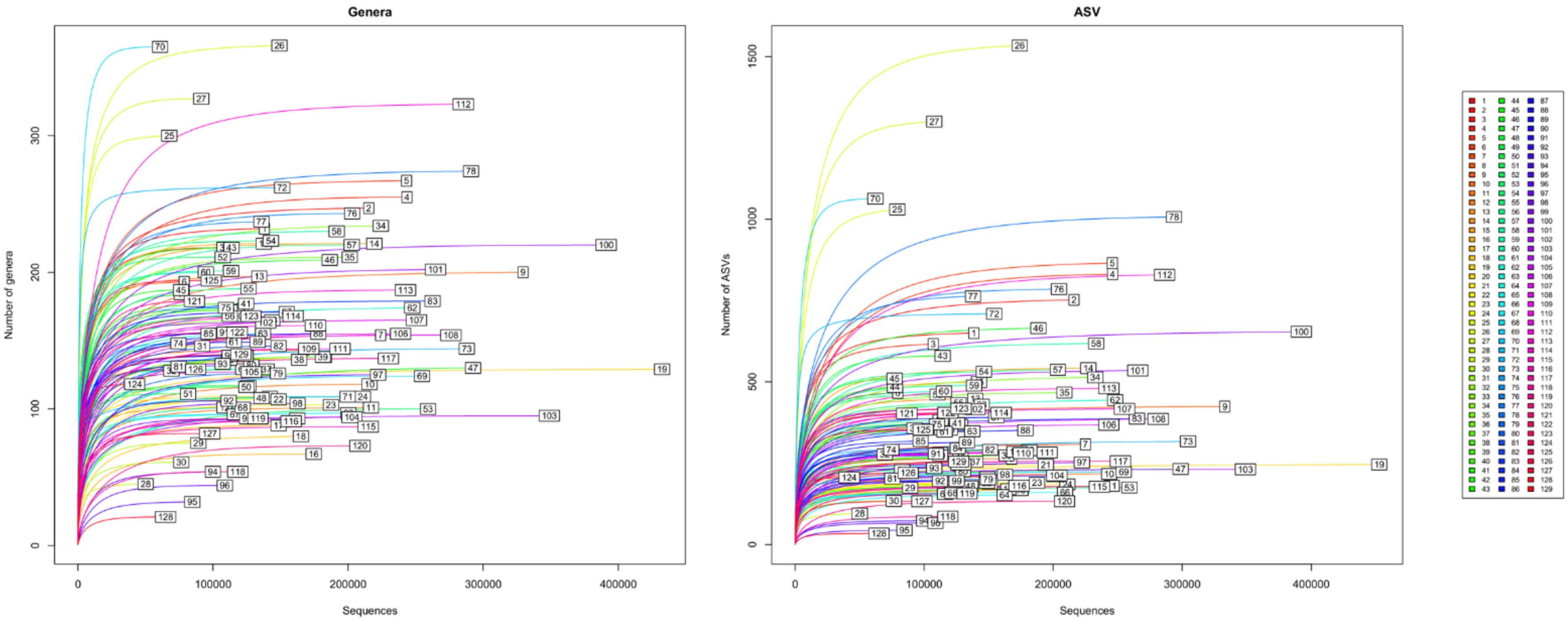

Figure S2. Rarefaction curves of the annotated sequences generated from Novaseq sequencing corresponding in genera and ASVs level.

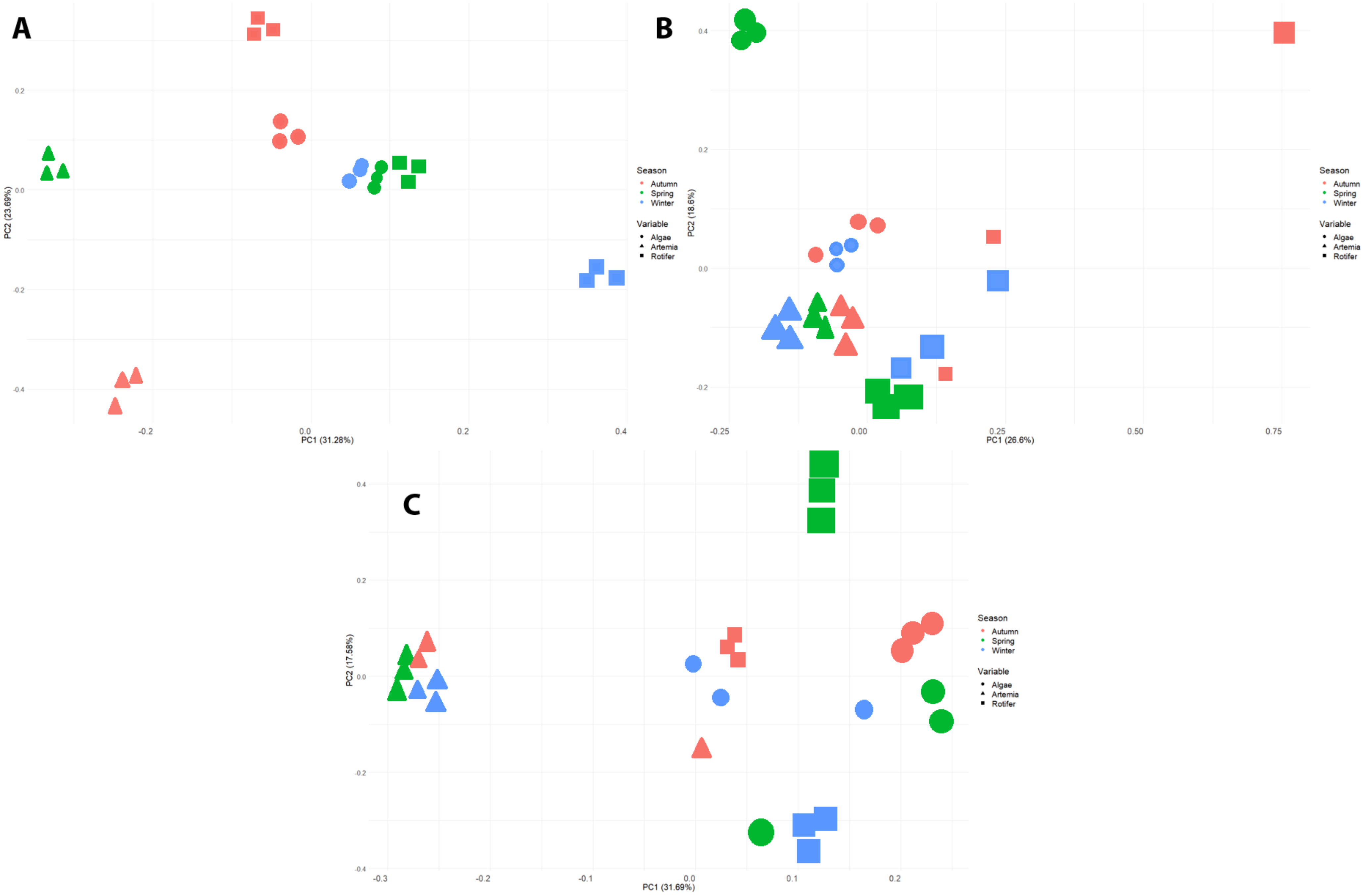
A
B
C
PC1 (31.28%)
PC2 (23.69%)
PC1 (26.6%)
PC2 (18.6%)
PC1 (31.69%)
PC2 (17.58%)
Season
Autumn
Spring
Winter
Variable
Algae
Artemia
Rotifer

Figure S3. Principal Component Analysis (PCA) of genera relative abundances in live feeds of A) HCMR hatchery, B) Hatchery A, C) Hatchery B. Samples have been grouped according to seasonal variability. Axis represent percentage of the major principal components.

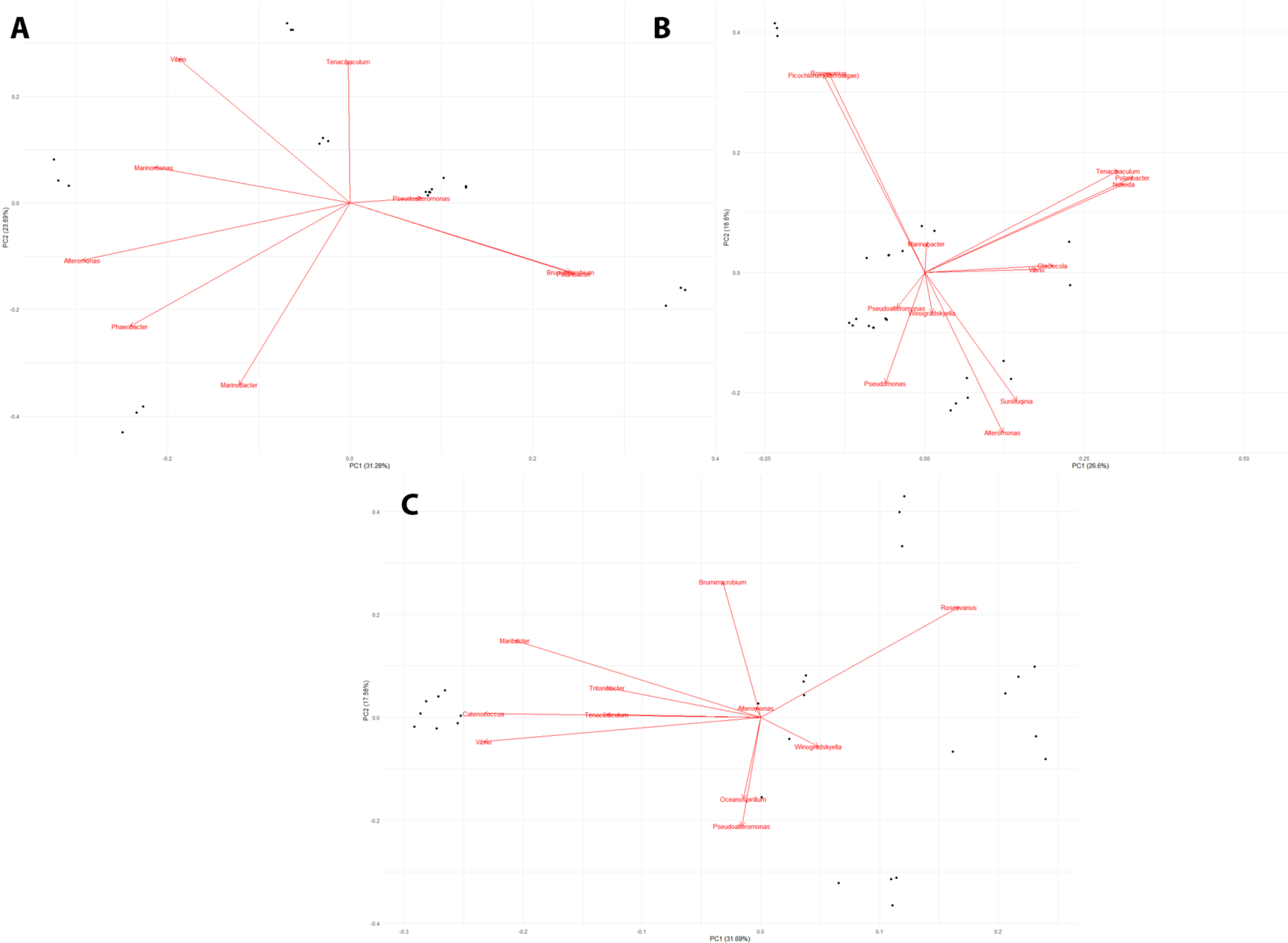
A
Vibrio
Tenacibaculum
Marinomonas
Pseudoalteromonas
Alteromonas
Phaeobacter
Marinobacter
PC1 (31.28%)
PC2 (23.69%)
B
Roseovarius
Picochlorum (Microalgae)
Tenacibaculum
Polaribacter
Nonlabens
Marinobacter
Glaciecola
Vibrio
Pseudoalteromonas
Winogradskyella
Pseudomonas
Sunxiuqinia
Alteromonas
PC1 (26.6%)
PC2 (18.6%)
C
Brumimicrobium
Roseovarius
Maribacter
Tritonibacter
Alteromonas
Catenococcus
Tenacibaculum
Vibrio
Winogradskyella
Oceanospirillum
Pseudoalteromonas
PC1 (31.69%)
PC2 (17.58%)

Figure S4. Loadings of PCAs from Figure S3**,** genera relative abundances in live feeds of A) HCMR hatchery, B) Hatchery A, C) Hatchery B. Samples have been grouped according to seasonal variability.

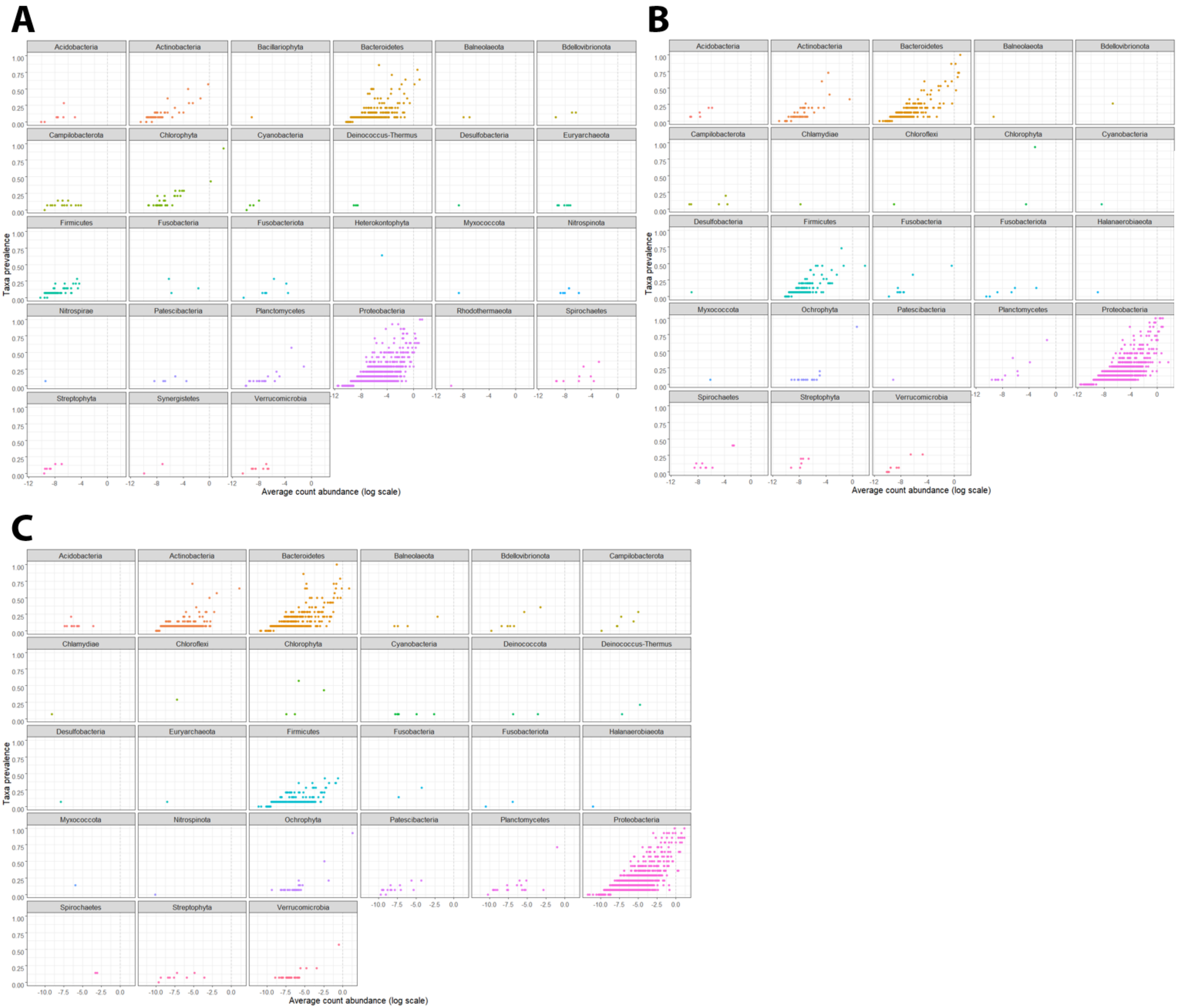
A
Acidobacteria
Actinobacteria
Bacillariophyta
Bacteroidetes
Balneolaeota
Bdellovibrionota
Campilobacterota
Chlorophyta
Cyanobacteria
Deinococcus-Thermus
Desulfobacteria
Euryarchaeota
Firmicutes
Fusobacteria
Fusobacteriota
Heterokontophyta
Myxococcota
Nitrospinota
Nitrospirae
Patescibacteria
Planctomycetes
Proteobacteria
Rhodothermaeota
Spirochaetes
Streptophyta
Synergistetes
Verrucomicrobia
Taxa prevalence
Average count abundance (log scale)
B
Acidobacteria
Actinobacteria
Bacteroidetes
Balneolaeota
Bdellovibrionota
Campilobacterota
Chlamydiae
Chloroflexi
Chlorophyta
Cyanobacteria
Desulfobacteria
Firmicutes
Fusobacteria
Fusobacteriota
Halanaerobiaeota
Myxococcota
Ochrophyta
Patescibacteria
Planctomycetes
Proteobacteria
Spirochaetes
Streptophyta
Verrucomicrobia
Taxa prevalence
Average count abundance (log scale)
C
Acidobacteria
Actinobacteria
Bacteroidetes
Balneolaeota
Bdellovibrionota
Campilobacterota
Chlamydiae
Chloroflexi
Chlorophyta
Cyanobacteria
Deinococcota
Deinococcus-Thermus
Desulfobacteria
Euryarchaeota
Firmicutes
Fusobacteria
Fusobacteriota
Halanaerobiaeota
Myxococcota
Nitrospinota
Ochrophyta
Patescibacteria
Planctomycetes
Proteobacteria
Spirochaetes
Streptophyta
Verrucomicrobia
Taxa prevalence
Average count abundance (log scale)

Figure S5. Phyla of most prevalent bacteria in A) HCMR hatchery, B) Hatchery A and C) Hatchery B. The relative abundances of the bacteria phylum are represented in a log scale.
