# Supplementary material for: Unveiling Emerging Opportunistic Fish Pathogens in Aquaculture: A Comprehensive Seasonal Study of Microbial Composition in Mediterranean Fish Hatcheries
Source: Microorganisms. 2024 Nov 10;12(11):2281. doi: 10.3390/microorganisms12112281 (PMC11596916; doi:10.3390/microorganisms12112281)
Supplement: Supplementary file 1 [file microorganisms-12-02281-s001.zip › Table S1.pdf]

Table S1. Grand Mean and Standard Error of Means (SEM) of relative abundances of the presumably opportunistic fish pathogenic species after our ASV blast analysis (first result) in HCMR hatchery among a) sampling point and b) among seasons. Different superscript letters indicate where the statistically significant difference occurs (One-way ANOVA, P≤0.05, Post-hoc test LSD; n/a for not available).

**HCMR_Hatchery**	**Inlet Water**	**Rotifer**	**Algae**	**Artemia**	**Outlet Water**			**Winter**	**Spring**	**Autumn**		
**Species**	Grand Mean	Grand Mean	Grand Mean	Grand Mean	Grand Mean	SEM	**P of sampling point**	Grand Mean	Grand Mean	Grand Mean	SEM	**P of seasons**
Aliivibrio fischeri	n/a	n/a	n/a	n/a	0.016	0.003	**0.011**	0.012	n/a	n/a	0.003	**0.017**
Photobacterium damselae	0.753	n/a	n/a	n/a	n/a	0.135	**0.014**	n/a	0.452	n/a	0.123	0.055
Vibrio alginolyticus	0.392^{a}	0.026^{b}	0.403^{a}	0.139^{a}	0.064^{b}	0.072	**0.023**	0.242	0.121	0.271	0.037	0.436
Vibrio anguillarum	0.059^{a}	4.215^{b}	0.004^{b}	0.001^{b}	1.449^{ab}	0.730	**0.022**	0.018	0.597	2.825	0.698	0.054
Vibrio atypicus	n/a	0.141^{a}	0.012^{b}	n/a	0.008^{b}	0.024	**0.020**	n/a	0.002	0.094	0.025	**0.027**
Vibrio bivalvicida	n/a	n/a	n/a	n/a	0.024	0.004	**0.000**	n/a	0.010	0.004	0.002	0.159
Vibrio brasiliensis	0.007^{a}	0.012^{a}	0.670^{a}	3.025^{b}	0.010^{a}	0.523	**0.000**	0.001	1.105	0.523	0.260	0.197
Vibrio campbellii	0.014^{a}	n/a	0.004^{ab}	0.002^{ab}	0.001^{b}	0.002	0.177	0.011^{a}	0.001^{b}	0.002^{ab}	0.003	0.110
Vibrio chagasii	0.283^{a}	0.005^{b}	0.003^{b}	n/a	0.028^{b}	0.049	**0.006**	0.021^{ab}	0.015^{a}	0.160^{b}	0.039	0.090
Vibrio cidicii	n/a	0.002	0.003	n/a	n/a	0.001	0.065	n/a	n/a	0.003	0.001	**0.002**
Vibrio coralliilyticus	0.005^{a}	0.007^{a}	0.875^{b}	0.694^{ab}	0.013^{b}	0.172	**0.015**	n/a	0.253	0.564	0.133	0.139
Vibrio cortegadensis	0.474^{ab}	0.048^{a}	0.008^{a}	n/a	1.057^{b}	0.183	0.039	0.789	0.004	0.316	0.186	0.062
Vibrio cyclitrophicus	0.001^{a}	n/a	n/a	n/a	0.049^{b}	0.009	**0.015**	0.037^{a}	n/a	0.001^{b}	0.010	**0.017**
Vibrio diabolicus	n/a	0.003^{a}	0.383^{b}	0.002^{a}	0.016^{a}	0.068	**0.015**	n/a	0.005^{a}	0.237^{b}	0.064	**0.047**

Vibrio europaeus	0.135	0.207	0.510	0.142	0.012	0.075	0.097	0.008[a]	0.170[ab]	0.399[b]	0.093	**0.035**
Vibrio fortis	0.159[ab]	0.001[a]	n/a	0.268[ab]	0.380[b]	0.067	**0.045**	0.382	0.126	n/a	0.092	**0.006**
Vibrio galatheae	0.066[a]c	0.012[b]	0.032[b]c	0.061c	0.036[ab]c	0.009	**0.012**	0.066[a]	0.042[a]	0.017[b]	0.011	**0.002**
Vibrio gallaecicus	n/a	n/a	n/a	n/a	0.096	0.017	**0.013**	0.072	n/a	n/a	0.019	**0.016**
Vibrio gallicus	0.012	n/a	0.001	0.154	n/a	0.027	**0.000**	n/a	0.034	0.036	0.009	0.285
Vibrio gigantis	0.217[a]	0.006[b]	n/a	n/a	0.050[b]	0.037	**0.024**	0.029	0.006	0.135	0.032	0.087
Vibrio hannami	0.007	n/a	n/a	n/a	n/a	0.001	**0.017**	0.005	n/a	n/a	0.001	**0.018**
Vibrio jasicida	0.024	n/a	n/a	n/a	n/a	0.004	**0.000**	0.014	0.004	n/a	0.003	0.052
Vibrio kanaloae	n/a	n/a	n/a	n/a	0.001	n/a	0.469	0.001	n/a	n/a	n/a	0.293
Vibrio lentus	n/a	n/a	0.006[a]	n/a	0.027[b]	0.005	**0.025**	0.020[a]	n/a	0.004[b]	0.005	**0.033**
Vibrio mediterranei	0.001	n/a	n/a	n/a	n/a	n/a	0.469	0.001	n/a	n/a	n/a	0.293
Vibrio neptunius	0.012[a]	0.303[a]	0.417[ab]	0.834[b]	0.016[a]	0.136	**0.014**	0.008	0.465	0.310	0.110	0.081
Vibrio ostreicida	n/a	n/a	n/a	n/a	0.004	0.001	**0.015**	n/a	0.003	n/a	0.001	0.057
Vibrio panuliri	n/a	n/a	n/a	0.003	n/a	0.001	**0.000**	n/a	0.001	n/a	n/a	0.060
Vibrio parahaemolyticus	0.007	0.009	0.009	0.013	0.013	0.001	0.845	0.024[a]	0.006[b]	0.002[b]	0.006	**0.000**
Vibrio pectenicida	0.078	n/a	n/a	n/a	n/a	0.014	**0.012**	0.058	n/a	n/a	0.016	**0.017**
Vibrio pelagius	0.005[a]	n/a	0.010[a]	0.040[b]	n/a	0.007	**0.001**	n/a	0.019	0.006	0.005	0.055
Vibrio proteolyticus	0.039[a]	n/a	n/a	0.138[b]	0.002c	0.024	**0.000**	0.001	0.047	0.032	0.011	0.131
Vibrio renipiscarius	0.002	n/a	n/a	n/a	n/a	n/a	**0.017**	0.001	n/a	n/a	n/a	**0.019**
Vibrio sagamiensis	0.019	n/a	n/a	n/a	n/a	0.003	**0.010**	n/a	0.011	n/a	0.003	0.060

Vibrio scophthalmi	n/a	0.064	n/a	n/a	0.003	0.011	**0.015**	0.002	n/a	0.038	0.010	0.064
Vibrio splendidus	n/a	n/a	n/a	n/a	0.001	n/a	0.109	n/a	n/a	n/a	n/a	0.077
Vibrio tapetis	0.170	n/a	n/a	n/a	n/a	0.030	**0.000**	n/a	0.056	0.046	0.014	0.305
Vibrio toranzoniae	n/a	0.002[a]	n/a	n/a	0.011[b]	0.002	**0.000**	0.002	0.004	0.002	0.001	0.533
Vibrio tubiashii	0.038[a]	0.001[a]	0.001[a]	1.332[b]	0.004[a]	0.236	**0.000**	0.001	0.511	0.047	0.133	0.080
Vibrio vulnificus	n/a	0.002	n/a	n/a	n/a	n/a	0.104	n/a	n/a	0.001	n/a	0.159
Vibrio xuii	0.049[a]	1.530[b]	0.107[a]	0.349[a]c	0.621c	0.241	**0.032**	0.041[a]	0.341[a]	1.151[b]	0.271	**0.025**
Vibrio zhuhaiensis	0.049	n/a	n/a	n/a	n/a	0.009	**0.015**	n/a	0.029	n/a	0.008	0.058
Tenacibaculum aestuarii	n/a	n/a	n/a	n/a	0.006	0.072	**0.023**	n/a	0.001	0.003	0.001	0.200
Tenacibaculum aestuariivivum	7.575[a]	0.008[b]	0.011[b]	0.044[b]	0.791[b]	1.324	**0.016**	5.668[a]	0.407[b]	0.107[b]	1.475	**0.021**
Tenacibaculum aiptasiae	0.002[a]	0.803[a]	0.003[a]	0.004[a]	2.440[b]	0.423	**0.012**	0.002[a]	0.110[a]	1.839[b]	0.486	**0.007**
Tenacibaculum ascidiaceicola	0.007[a]	0.008[a]	0.001[a]	n/a	0.005[b]	0.001	0.178	0.005	0.007	0.001	0.001	0.142
Tenacibaculum caenipelagi	n/a	n/a	n/a	n/a	0.001	n/a	0.469	n/a	n/a	n/a	n/a	0.417
Tenacibaculum holothuriorum	0.017	n/a	n/a	n/a	n/a	0.003	**0.015**	n/a	0.010	n/a	0.003	0.057
Tenacibaculum insulae	0.006[a]	n/a	n/a	n/a	0.029[b]	0.005	**0.000**	n/a	0.005	0.016	0.004	**0.042**
Tenacibaculum litopenaei	0.001	n/a	n/a	n/a	n/a	n/a	0.105	0.001	n/a	n/a	n/a	0.075
Tenacibaculum lutimaris	n/a	n/a	n/a	n/a	0.004	0.001	**0.004**	n/a	0.002	n/a	0.001	0.079
Tenacibaculum mesophilum	0.003	n/a	n/a	0.004	0.007	0.001	0.093	n/a	0.008	n/a	0.002	**0.000**
Tenacibaculum ovolyticum	n/a	n/a	n/a	n/a	n/a	n/a	0.469	n/a	n/a	n/a	n/a	0.417

Tenacibaculum skagerrakense	0.004	n/a	n/a	n/a	n/a	0.001	0.105	n/a	0.003	n/a	0.001	0.160
Tenacibaculum soleae	0.010	n/a	n/a	n/a	n/a	0.002	**0.014**	n/a	n/a	0.006	0.002	0.056
Tenacibaculum adriaticum	0.156[a]	0.150[a]	0.348[ab]	n/a	0.912[b]	0.143	**0.031**	0.840[a]	0.102[b]	0.166[b]	0.193	**0.004**
