# Supplementary material for: Unveiling Emerging Opportunistic Fish Pathogens in Aquaculture: A Comprehensive Seasonal Study of Microbial Composition in Mediterranean Fish Hatcheries
Source: Microorganisms. 2024 Nov 10;12(11):2281. doi: 10.3390/microorganisms12112281 (PMC11596916; doi:10.3390/microorganisms12112281)
Supplement: Supplementary file 1 [file microorganisms-12-02281-s001.zip › Table S2.pdf]

Table S2. Grand Mean and Standard Error of Means (SEM) of relative abundances of the presumably opportunistic fish pathogenic species after our ASV blast analysis (first result) in Hatchery A among a) sampling point and b) among seasons. Different superscript letters indicate where the statistically significant difference occurs (One-way ANOVA, P≤0.05, Post-hoc test LSD; n/a for not available).

**Hatchery A**	**Inlet Water**	**Rotifer**	**Algae**	**Artemia**	**Outlet Water**			**Winter**	**Spring**	**Autumn**		
**Species**	Grand Mean	Grand Mean	Grand Mean	Grand Mean	Grand Mean	SEM	**P of sampling point**	Grand Mean	Grand Mean	Grand Mean	SEM	**P of seasons**
Aliivibrio fischeri	0.015	n/a	n/a	n/a	0.006	0.003	0.515	n/a	0.009	0.004	0.002	0.524
Photobacterium damselae	0.097	0.002	n/a	n/a	0.093	0.021	0.562	n/a	0.058	0.058	0.016	0.597
Tenacibaculum aestuariivivum	0.340[a]	n/a	0.014[b]	0.031[b]	0.096	0.057	0.064	0.272[a]	0.015[b]	0.002[b]	0.072	**0.012**
Tenacibaculum aiptasiae	0.009[a]	n/a	n/a	0.001[a]	0.028[b]	0.005	**0.000**	0.001	0.011	0.010	0.003	0.193
Tenacibaculum ascidiaceicola	0.006[a]	n/a	n/a	n/a	0.014[b]	0.002	**0.001**	n/a	0.009	0.004	0.002	**0.030**
Tenacibaculum caenipelagi	n/a	n/a	n/a	n/a	0.004	0.001	**0.032**	0.003	n/a	n/a	0.001	0.080
Tenacibaculum lutimaris	n/a	n/a	n/a	n/a	0.001	0.000	**0.028**	0.001	n/a	n/a	0.000	0.073
Tenacibaculum mesophilum	0.003[a]	n/a	0.003[a]	0.026[b]	n/a	0.004	**0.017**	0.002	0.002	0.016	0.004	0.071
Tenacibaculum sediminilitoris	0.015[a]	n/a	n/a	n/a	0.051[b]	0.009	**0.000**	n/a	0.026	0.013	0.006	0.071
Tenacibaculum soleae	0.003[a]	n/a	n/a	n/a	0.008[b]	0.001	**0.000**	0.001	0.004	0.002	0.001	0.176
Tenacibaculum adriaticum	0.637[a]	0.002[b]	0.006[b]	0.001[b]	0.218[b]	0.110	**0.025**	0.345	0.116	0.058	0.072	0.270
Vibrio alfacsensis	0.052	n/a	n/a	n/a	0.062	0.013	0.561	n/a	0.031	0.037	0.009	0.605
Vibrio alginolyticus	0.236[a]	0.138[a]	0.031[a]	14.365[b]	0.307[a]	2.538	**0.000**	4.985	3.567	0.494	1.082	0.218
Vibrio anguillarum	0.003	0.002	n/a	n/a	0.006	0.001	0.081	0.003	0.002	0.001	0.001	0.452

Vibrio areninigrae	0.001^{a}	0.378^{b}	n/a	n/a	0.001^{a}	0.068	**0.000**	0.074	0.100	0.054	0.011	0.747
Vibrio atypicus	0.082^{a}c	0.005^{b}	0.009^{ab}	0.093c	0.014^{ab}	0.017	**0.046**	0.043	0.016	0.063	0.011	0.319
Vibrio azureus	n/a	n/a	n/a	n/a	0.001	0.000	0.419	0.001	n/a	n/a	0.000	0.376
Vibrio barjaei	n/a	n/a	0.002^{a}	0.011^{b}	n/a	0.002	**0.001**	0.001	0.002	0.005	0.001	0.222
Vibrio brasiliensis	0.022^{a}	0.003^{a}	0.002^{a}	0.350^{b}	0.017^{a}	0.061	**0.000**	0.132	0.083	0.021	0.026	0.236
Vibrio campbellii	0.004	n/a	n/a	0.006	0.002	0.001	0.107	0.003	0.005	n/a	0.001	0.113
Vibrio chagasii	0.041^{a}	5.702^{b}	0.003^{a}	0.064^{a}	0.053^{a}	1.013	**0.000**	1.298	1.433	0.787	0.161	0.766
Vibrio coralliilyticus	0.011^{a}	n/a	0.003^{a}	0.061^{b}	0.020^{a}	0.010	**0.000**	0.019	0.018	0.021	0.001	0.971
Vibrio cortegadensis	0.005^{a}	0.005^{a}	0.005^{a}	0.004^{a}	0.015^{b}	0.002	**0.043**	0.010	0.006	0.004	0.001	0.195
Vibrio cyclitrophicus	n/a	1.838^{a}	0.001^{b}	n/a	0.001^{b}	0.329	**0.000**	0.430	0.448	0.227	0.058	0.722
Vibrio diabolicus	0.003^{a}	0.001^{a}	0.001^{a}	0.591^{b}	0.002^{a}	0.105	**0.000**	0.124	0.080	0.192	0.026	0.539
Vibrio europaeus	0.013	n/a	n/a	0.003	0.016	0.003	0.170	0.001	0.012	0.007	0.002	0.329
Vibrio fortis	0.415^{a}	0.002^{a}	0.005^{a}	0.054^{a}	0.955^{b}	0.165	**0.002**	0.031^{a}	0.599^{b}	0.228^{b}	0.136	**0.040**
Vibrio galatheae	0.417^{a}	0.031^{a}	0.100^{a}	10.023^{b}	0.651^{a}	1.742	**0.000**	3.104	2.519	1.110	0.483	0.462
Vibrio gallicus	0.015^{a}	n/a	0.006^{a}	0.433^{b}	0.016^{a}	0.076	**0.000**	0.080	0.067	0.145	0.020	0.505
Vibrio gigantis	0.004^{a}	0.006^{a}	n/a	n/a	0.078^{b}	0.014	**0.004**	0.045	0.005	0.003	0.011	0.053
Vibrio hannami	0.002^{ab}	n/a	n/a	0.001^{a}	0.006^{b}	0.001	**0.022**	n/a	0.004^{a}	0.001^{b}	0.001	**0.007**
Vibrio hepatarius	0.002	n/a	n/a	n/a	n/a	0.000	0.419	n/a	0.001	n/a	0.000	0.376
Vibrio jasicida	0.004	n/a	n/a	n/a	0.005	0.001	0.562	n/a	0.003	0.003	0.001	0.607
Vibrio kanaloae	n/a	0.024^{a}	n/a	n/a	0.161^{b}	0.028	**0.014**	0.101^{a}	0.008^{b}	0.002^{b}	0.026	**0.042**

Vibrio lentus	n/a	0.004[a]	n/a	n/a	0.303[b]	0.054	**0.011**	0.184[a]	0.001[b]	n/a	0.050	**0.044**
Vibrio mediterranei	n/a	n/a	0.005[a]	0.039[b]	n/a	0.007	**0.000**	0.006	0.006	0.014	0.002	0.402
Vibrio mytili	0.001	n/a	n/a	n/a	0.002	0.000	0.543	n/a	0.001	0.001	0.000	0.572
Vibrio neptunius	0.002[a]	0.055[a]	0.015[a]	0.363[b]	0.010[a]	0.062	**0.000**	0.045	0.046	0.176	0.035	0.140
Vibrio parahaemolyticus	0.025[a]	0.013[a]	0.031[a]	7.836[b]	0.039[a]	1.397	**0.000**	2.659	1.858	0.250	0.578	0.229
Vibrio pelagius	0.181[a]	0.060[a]	0.002[a]	0.024[a]	0.407[b]	0.067	**0.003**	0.021[a]	0.270[b]	0.113[ab]	0.059	**0.032**
Vibrio proteolyticus	0.071[a]	0.125[a]	0.029[a]	0.916[b]	0.049[a]	0.152	**0.000**	0.141	0.145	0.427	0.077	0.194
Vibrio renipiscarius	0.003	n/a	n/a	n/a	n/a	0.000	0.419	n/a	0.002	n/a	0.000	0.376
Vibrio sagamiensis	0.003[a]	n/a	0.002[a]	0.076[b]	0.009[a]	0.013	**0.000**	0.011	0.013	0.029	0.005	0.340
Vibrio scophthalmi	0.090	n/a	n/a	n/a	0.107	0.022	0.562	n/a	0.054	0.064	0.016	0.606
Vibrio splendidus	n/a	0.594	n/a	n/a	n/a	0.106	**0.000**	0.132	0.154	0.070	0.021	0.683
Vibrio tapetis	0.068	0.009	n/a	n/a	0.094	0.018	0.530	0.007	0.043	0.053	0.012	0.666
Vibrio toranzoniae	n/a	0.826[a]	n/a	n/a	0.001[b]	0.148	**0.000**	0.188	0.197	0.112	0.022	0.792
Vibrio tubiashii	0.015[a]	n/a	n/a	0.053[b]	0.031[ab]	0.009	**0.000**	0.021	0.029	0.010	0.005	0.217
Vibrio vulnificus	n/a	n/a	n/a	0.006	n/a	0.001	**0.014**	n/a	n/a	0.003	0.001	0.057
Vibrio xuii	0.084[a]	1.945[b]	0.038[a]	1.321c	0.258[a]	0.343	**0.000**	0.795	0.751	0.642	0.037	0.894
