# Supplementary material for: Unveiling Emerging Opportunistic Fish Pathogens in Aquaculture: A Comprehensive Seasonal Study of Microbial Composition in Mediterranean Fish Hatcheries
Source: Microorganisms. 2024 Nov 10;12(11):2281. doi: 10.3390/microorganisms12112281 (PMC11596916; doi:10.3390/microorganisms12112281)
Supplement: Supplementary file 1 [file microorganisms-12-02281-s001.zip › Table S3.pdf]

Table S3. Grand Mean and Standard Error of Means (SEM) of relative abundances of the presumably opportunistic fish pathogenic species after our ASV blast analysis (first result) in Hatchery B among a) sampling point and b) among seasons. Different superscript letters indicate where the statistically significant difference occurs (One-way ANOVA, P≤0.05, Post-hoc test LSD; n/a for not available).

**Hatchery B**	**Inlet Water**	**Rotifer**	**Algae**	**Artemia**	**Outlet Water**			**Winter**	**Spring**	**Autumn**		
**Species**	Grand Mean	Grand Mean	Grand Mean	Grand Mean	Grand Mean	SEM	**P of sampling point**	Grand Mean	Grand Mean	Grand Mean	SEM	**P of seasons**
Tenacibaculum aestuarii	n/a	n/a	n/a	0.002	n/a	0.000	**0.021**	0.001	n/a	n/a	0.000	0.306
Tenacibaculum aestuariivivum	0.585^{a}	0.001^{a}	0.002^{a}	0.002^{a}	1.693^{b}	0.294	**0.000**	0.685	0.161	0.508	0.126	0.287
Tenacibaculum aiptasiae	0.009	n/a	n/a	n/a	0.032	0.006	**0.000**	0.013	0.001	0.011	0.003	0.121
Tenacibaculum ascidiaceicola	0.003	0.001	n/a	n/a	0.003	0.001	0.327	n/a	0.002	0.001	0.001	0.321
Tenacibaculum caenipelagi	0.004^{a}	n/a	n/a	n/a	0.016^{b}	0.003	**0.000**	0.006	n/a	0.006	0.002	0.133
Tenacibaculum litopenaei	0.131	n/a	n/a	n/a	n/a	0.023	**0.016**	n/a	0.052	n/a	0.014	0.186
Tenacibaculum lutimaris	n/a	n/a	n/a	n/a	n/a	0.000	0.265	n/a	n/a	n/a	0.000	0.518
Tenacibaculum mesophilum	0.016^{a}	0.001^{a}	0.003^{a}	1.280^{b}	n/a	0.228	**0.000**	0.315	0.255	0.260	0.016	0.954
Tenacibaculum sediminilitoris	0.213	n/a	n/a	n/a	0.192	0.044	**0.000**	0.092	0.059	0.062	0.008	0.796
Tenacibaculum soleae	0.007	n/a	n/a	n/a	0.021	0.004	**0.000**	0.010	n/a	0.006	0.002	0.097
Tenacibaculum adriaticum	1.212^{a}	n/a	0.897^{ab}	0.015^{b}	0.140^{ab}	0.225	0.074	0.568	0.527	0.027	0.142	0.374
Vibrio aestuarianus	n/a	n/a	n/a	n/a	n/a	0.000	0.469	n/a	n/a	n/a	0.000	0.293
Vibrio alginolyticus	1.972^{a}	1.194^{a}	0.083^{a}	16.607^{b}	0.397^{a}	2.823	**0.000**	4.050	4.714	3.741	0.234	0.938
Vibrio anguillarum	0.007	0.006	0.001	0.005	0.016	0.002	0.176	0.014^{a}	0.003^{b}	0.002^{b}	0.003	**0.021**

Vibrio atypicus	0.074	n/a	0.013	0.020	0.006	0.012	0.088	0.002	0.029	0.027	0.007	0.336
Vibrio barjaei	0.331	n/a	0.002	0.005	n/a	0.059	0.203	0.132	n/a	0.005	0.035	0.429
Vibrio brasiliensis	0.006[a]	n/a	0.020[b]	0.005[b]	0.062[b]	0.010	**0.037**	0.012	0.038	0.005	0.008	0.171
Vibrio campbellii	n/a	0.107	n/a	0.993	n/a	0.174	**0.000**	0.208	0.247	0.255	0.012	0.958
Vibrio chagasii	0.159[a]	0.014[a]	0.004[ab]	0.022[a]	0.257[b]	0.045	**0.000**	0.098	0.073	0.088	0.006	0.874
Vibrio coralliilyticus	0.009	0.002	0.002	0.005	0.017	0.002	0.088	0.001	0.013	0.007	0.003	0.056
Vibrio cortegadensis	0.464[a]	0.002[b]	n/a	0.002[b]	1.082[a]	0.190	**0.000**	0.432	0.098	0.385	0.085	0.279
Vibrio cyclitrophicus	0.062[a]	n/a	0.001[b]	0.059[a]	0.087[a]	0.016	**0.000**	0.046	0.034	0.042	0.003	0.825
Vibrio diabolicus	0.669[a]	n/a	n/a	0.057[b]	0.002[b]	0.117	**0.003**	n/a	0.268	0.044	0.068	0.141
Vibrio diazotrophicus	n/a	0.426	n/a	n/a	n/a	0.076	**0.014**	n/a	n/a	0.320	0.087	**0.016**
Vibrio europaeus	0.002	n/a	n/a	n/a	0.008	0.001	**0.000**	0.004	n/a	0.002	0.001	0.090
Vibrio fluvialis	n/a	0.005	n/a	n/a	n/a	0.001	**0.015**	n/a	n/a	0.003	0.001	**0.017**
Vibrio fortis	1.074	0.021	1.699	n/a	0.030	0.313	0.470	0.015	1.450	0.019	0.390	0.183
Vibrio galatheae	0.177[a]	1.810[b]	0.160[a]	11.699c	0.179[a]	2.009	**0.000**	3.357	2.973	2.563	0.187	0.923
Vibrio gallaecicus	0.057[a]	n/a	n/a	n/a	0.128[b]	0.023	**0.000**	0.047	0.012	0.051	0.010	0.301
Vibrio gallicus	0.401[a]	n/a	0.008[b]	0.461[a]	0.024[b]	0.093	**0.000**	0.096	0.260	0.126	0.041	0.292
Vibrio gigantis	n/a	0.001	n/a	0.014	0.001	0.002	**0.009**	0.005	0.004	0.001	0.001	0.567
Vibrio hannami	n/a	0.002	n/a	n/a	n/a	0.000	0.110	n/a	n/a	0.002	0.000	0.078
Vibrio jasicida	0.019	n/a	n/a	n/a	0.008	0.003	0.082	0.004	0.006	0.003	0.001	0.858
Vibrio kanaloae	0.447[a]	n/a	n/a	n/a	0.117[b]	0.077	**0.001**	0.050	0.168	0.039	0.034	0.310

Vibrio lentus	0.812[a]	n/a	0.001[b]	0.007[b]	0.158[ab]	0.140	**0.001**	0.064	0.314	0.058	0.069	0.231
Vibrio litoralis	0.002	n/a	n/a	n/a	0.006	0.001	**0.000**	0.003	n/a	0.002	0.001	0.187
Vibrio mediterranei	n/a	n/a	0.009	0.014	n/a	0.003	0.509	n/a	n/a	0.017	0.005	**0.048**
Vibrio neptunius	0.015	0.008	0.023	0.082	0.080	0.015	0.439	0.003	0.057	0.076	0.018	0.186
Vibrio pacinii	0.139[a]	n/a	n/a	n/a	0.484[b]	0.084	**0.000**	0.175	0.021	0.188	0.044	0.219
Vibrio parahaemolyticus	0.052[a]	0.074[aa]	0.031	0.629[b]	0.069[a]	0.103	**0.000**	0.142	0.147	0.266	0.033	0.370
Vibrio pectenicida	0.086	n/a	n/a	n/a	n/a	0.015	0.105	n/a	0.035	n/a	0.009	0.334
Vibrio pelagius	0.144	0.009	0.606	0.003	0.004	0.104	0.504	n/a	0.424	0.009	0.114	0.309
Vibrio plantisponsor	n/a	0.053	n/a	n/a	n/a	0.009	**0.015**	n/a	n/a	0.040	0.011	**0.016**
Vibrio proteolyticus	0.482[a]	n/a	0.040[b]	0.644[a]	0.067[b]	0.118	**0.000**	0.124	0.317	0.254	0.047	0.400
Vibrio renipiscarius	n/a	n/a	n/a	n/a	0.001	0.000	**0.015**	n/a	0.001	n/a	0.000	0.057
Vibrio sagamiensis	0.093[a]	n/a	0.001[b]	0.016[ab]	0.021[ab]	0.015	**0.014**	0.011	0.038	0.014	0.007	0.406
Vibrio scophthalmi	n/a	0.061	n/a	n/a	n/a	0.011	**0.000**	n/a	0.010[a]	0.033[b]	0.008	**0.050**
Vibrio splendidus	n/a	n/a	n/a	n/a	0.001	0.000	0.218	0.001	n/a	n/a	0.000	0.527
Vibrio tapetis	n/a	n/a	n/a	n/a	0.005	0.001	0.148	n/a	n/a	0.004	0.001	0.100
Vibrio toranzoniae	n/a	n/a	n/a	0.012	n/a	0.002	**0.000**	0.002	0.002	0.004	0.001	0.714
